# An expression of uncertainty and its application to positioning: a quality-metric and optimal ranges for the identification of cells with RFID

**DOI:** 10.1186/s40064-015-1084-6

**Published:** 2015-07-24

**Authors:** Eduardo Del Rio, Luiz Felipe Ferreira

**Affiliations:** Geodesy Laboratory, Department of Geophysics, Graduate School of Science, Kyoto University, Kyoto, Japan; Instituto Militar de Engenharia, Seção de Engenharia Cartográfica, SE/6, Rio de Janeiro, Brazil; Pontifícia Universidade Católica do Rio de Janeiro, Programa de Pós-graduação em Metrologia para a Qualidade e Inovação, Rio de Janeiro, Brazil

**Keywords:** Uncertainty, Propagation of uncertainties, Positioning, Error modeling, Cell-id, RFID, Optimization, Optimal range

## Abstract

Here we devise an approach to model error and its propagation. Without approximations, we define the uncertainty of a measurement as its maximum possible error (*maper*). Thus, we propose and solve analytically two optimization problems. The one designed to determine the uncertainty of a measurement, the other specifically designed to optimize the accuracy of a RFID location system. The usefulness of this general approach is shown by applying it to the particular instance of estimating the coordinates of a person in real-time using RFID devices. This way, exact formulae to evaluate the quality of this measurement are mathematically deduced, which is useful, for example, to predict whether an inexpensive RFID location technology can meet a desired quality standard or not. The second optimization problem proposed here defines an optimal range (*orange*) for the RFID devices employed. Again, analytically, its exact formulae were derived. We propose an approach to distribute RFID tags for a positioning system based solely on RFID technology. In the light of the formulae, its quality is good enough as to locate emergency phone calls in real time. We found that key to an optimal performance is the range used and the distance between consecutive tags.

**Electronic supplementary material:**

The online version of this article (doi:10.1186/s40064-015-1084-6) contains supplementary material, which is available to authorized users.

## Background

### Major concerns

On the one side, this paper concerns with expressing uncertainty. A general expression of uncertainty can be used in any scientific field, but especially for the one concerned here (geodesy and navigation) inasmuch as the standard uncertainty by means of standard deviation (SD) is helpless to study the problem under scrutiny. In fact, for continuous measurements based on identification of cells (Cell-Id) such as real time tracking of goods and people, if the uncertainty is large—as is the case here—the measurand—quantity being measured—remains constant over long periods of time which yields null deviations from the mean and thus a null SD. As the uncertainty is known to be large this null sd has evidently nothing to do with a highly accurate output data.

On the other side, this paper concerns with the real time tracking of goods and people. Much has been developed in this area over the past decades especially with the advent of the Global Navigation Satellite Systems (GNSS), from which GPS is its forefront. But these GNSS exhaust not the needs for tracking of goods. In this regard, several alternatives have been studied, some GNSS-free others to work integrated with it. Notwithstanding, these still exhaust not these needs. Among the existing alternatives the one eyed for herein is RFID-based (acronym for radio-frequency identification).

**Fig. 1:**
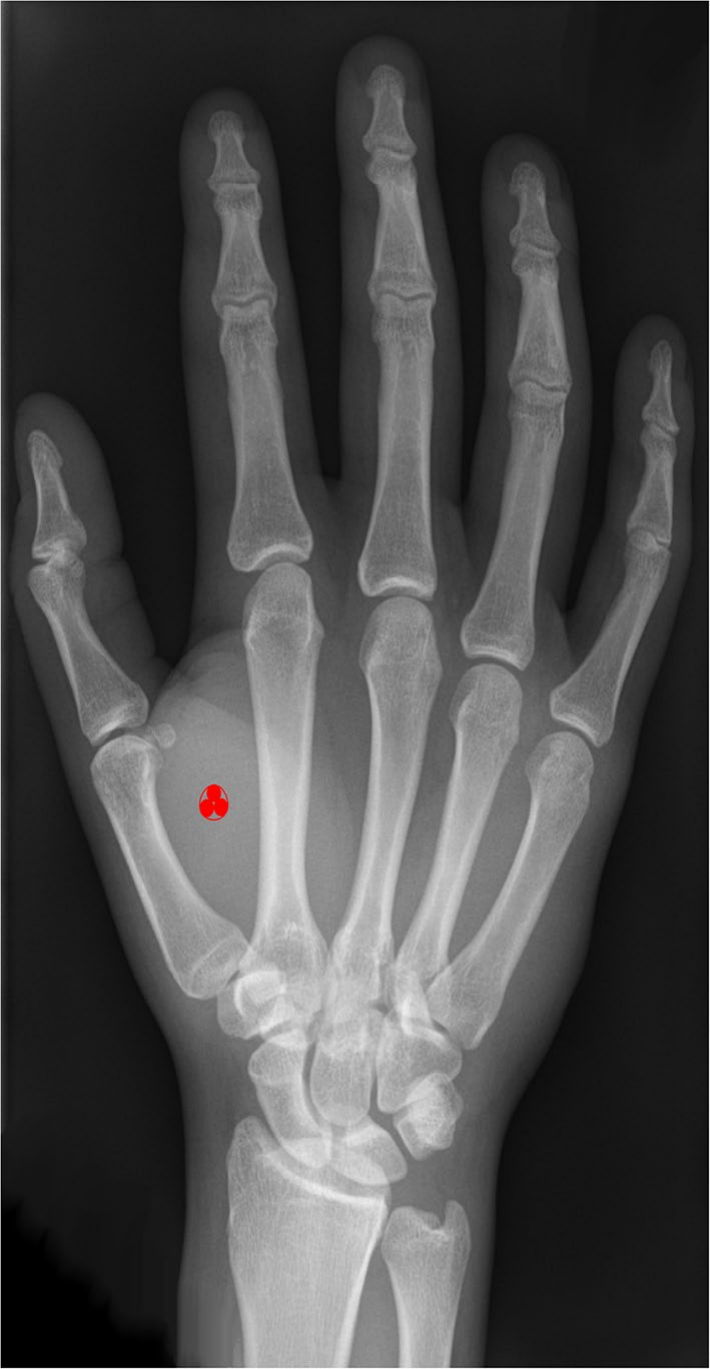
RFID tag implantations in the right hand. People can already receive a mark on their right hand by means of an RFID tag implanted in it (*red mark*). Picture originally published under CCAL 3.0 at http://en.wikipedia.org/wiki/Hand.

RFID is a technology devised to substitute bar codes and its recent development includes human implants in its scope, Fig. 1 and Peterson (2001). These implants are to enable an unambiguous identification of its host. This way, a society free of printed money bills could emerge as each and every financial transaction is done electronically by credit or debit cards (Peterson 2001). The implications of the advent of such universal credit system and its foreshadowing of an Orwellian totalitarian state (Orwell 1949) are the object of an investigation in itself and are not in the scope of this paper, but certainly they constitute but a driving force behind its inception.

Noteworthy, the application of RFID devices done throughout the paper is not concerned with implantable tags. We will concern with active tags that are to be attached to an object. But the application by human implants has an importance in that it served as a motivation for the choice of the research topic that concerns to the current paper.

### Uncertain science

Science is still limited on modeling nature and handling uncertainties (Palmer and Hardaker 2011). A problem is that all sources of uncertainty cannot be eliminated and the approaches available to handle it are each tangled by their own limitations (BIPM 2008).

Uncertainty in science remains and as political decisions can be influenced by scientific advice, this uncertainty may propagate over policies and in the end affect citizens other than scientists. Some examples are the following: the current debate on climate change (Smith and Stern 2011; Smith 2011; Slingo 2011), whether or to which extent it is anthropogenic, how will this change evolve over the next years and what should we do about it; macroeconomic policy-making (Aikman 2011); health policy-making and clinical practice (Wells et al. 2011).

Therefore, advances on handling uncertainty in science have the potential to outstrip academia, as lay persons are also benefited.

### Unreasonable uncertainty evaluations

The quality evaluation of measurements is a fundamental problem to any field in which a quantity has to be measured. Direct measurements are usually evaluated by the standard uncertainty (BIPM 2008)—i.e., the standard deviation (SD). For quantities measured in terms of a set of input quantities, a common approach is to evaluate their measurement by the law of propagation of uncertainties, for details refer to BIPM (2008), Kacker et al. (2007). However, this law is generally used in its simplified form, which assumes a first-order Taylor series approximation of the measurand function (i.e., that the behavior of the quantity being measured is well described by a linear function) and that the input quantities are uncorrelated [this simplified formula is traditionally referred to as law of error propagation, a misnomer (Kacker et al. 2007)]. If higher order approximations are to be included, restrictive assumptions have to be made (Kacker et al. 2007). Meanwhile, the major reason for unreasonable uncertainty evaluations is the non-identification and, consequently, non-incorporation of the correlation between the input quantities (Kacker et al. 2007).

On the other side, to determine the propagation of uncertainties when the measurand function is non-linear, a routine is to use a Monte Carlo simulation (BIPM 2008) to generate a probability density function (pdf) for the measurand from propagations of the pdf assigned to the input quantities. However, this probabilistic approach (i.e., distinct simulations of the same input data set may yield distinct output data) needs a pdf for the input quantities, and it also requires the joint distribution of correlated input variables, which is often unknown or difficult to simulate (Kacker et al. 2007).

### We cannot locate everyone ubiquitously yet, but what if we could?

Ubiquitous positioning is becoming feasible as diverse location technologies (Bensky 2008; Groves 2008; Do et al. 2007; De Lorenzo 2009) are being created. Designed to give the position of any point in the Earth at any given epoch, GPS is the most important (Bensky 2008; Groves 2008) location technology nowadays. However, when it comes to indoor and urban environments, because of the non-line-of-sight (Bensky 2008; Groves 2008; Do et al. 2007; Prost 2008) (NLOS) between satellites and receivers, the quality of the estimated position can be severely degraded. Moreover, these are precisely the regions where ubiquitous location would be of greater interest, for the majority of the Earth’s inhabitants dwell in urban zones—and its indoor regions—(CIA 2011), and consequently, their mobile terminals—including cell-phones—lie most of the time. Therefore, to locate a person or an object anywhere, at any time is still out of reach.

The ubiquitous positioning of each person may be used in several ways. Location-based services (Kupper 2005) as mobile marketing, mobile gaming, mobile yellow pages and enhanced emergency services become available, fostering profit generation and safety of life. Likewise, improved human-centric sensing (Srivastava et al. 2012), and in particular, deeper studies of individual human mobility patterns (Gonzalez et al. 2008) based on a more accurate and continuous tracking of a great number of people would be feasible. Hence, contributions to the knowledge about the laws governing human motion are likely to be made, which may lead to an understanding of the spread of biological (Eubank et al. 2004) and mobile (Kleinberg 2007) viruses. This, in turn, could become a step towards forecasting the spread of pandemic diseases in a regional and a global scale.

### About this article

As science fails to measure all quantities without uncertainty (BIPM 2008), we turn to the realm of mathematics which is itself the closest that men alone can get to certainty. In this sense, to build our own analytic (i.e., without approximations by series expansions) and deterministic (i.e., non-probabilistic) approach to error modeling, we define a concept and we express it mathematically, Eq. (1). This enables an evaluation of a measurement’s quality in the light of this general concept as we show its usefulness by applying it to the particular instance of estimating the position (i.e., the coordinates) of a person in real-time using wireless positioning techniques based on RFID technology. We also present an approach to distribute RFID tags over indoor and urban areas—the regions where GPS performs worse—and we show how our error model can be used to study its performance through numerical experiments.

RFID is not a new technology, since its origin dates back to the Second World War (Glover and Batt 2006). But, as a consequence of cost and size (Catuto et al. 2010; Peterson 2001; Zabow et al. 2008) reductions, RFID is becoming increasingly used in our society, especially when it works as an upgrade to bar codes (Gebbers and Adamchuk 2010; EC 2008). We divide the RFID devices into two categories: tags and readers. An RFID tag is a small radio-frequency transmitting device that sends a signal which covers a certain region, with a known latency (time delay between consecutive signal transmissions) and with an identifying code corresponding to it (this definition corresponds to active tags which have their own source of energy, typically batteries). Occasionally, the tag’s memory will be able to store data other than its unique identity, which might also be transmitted through its signal. The signal and its code are readable by an RFID reader, which can also be a small device (i.e., small enough to be carried by a person, like a cell-phone). Moreover, along a given direction, the distance from the RFID tags in which their signal can be detected by the reader is called range. Basically, to build an error propagation model, we will first assume that the region covered by the tags signal is a sphere; afterwards, we will employ this spherical range model to determine the error propagation when any generic range surface is considered. The range of RFID tags varies (Glover and Batt 2006) from dozens of centimeters to tens or even hundreds of meters.

The positioning techniques considered in this article are aimed at the real-time localization of a person (i.e., the user). Without loss of generality, we consider that the user has an RFID reader and that he has to locate himself based solely on it and on the coordinates of RFID tags nearby (this RFID reader is not used to measure distances), where each tag is attached to a point with a known location (this is known as a handset-based approach). It should be clear that there are not time, angles, received signal strength indications (RSSI) or any other quantities being measured. This way, we consider the simplest possible techniques to locate the user in real time and to draw our conclusions.

## Results

### Defining the concept

The accuracy of a measurement is defined as: “closeness of the agreement between the result of a measurement and a true value of the measurand” (BIPM 2008). In general, we don’t know the true value of a quantity (i.e., the measurand), and, as in the particular case of an RFID-based positioning approach, we have infinite possibilities for it. Moreover, despite being often expressed by a multiple of the unit of measurement concerned, the accuracy is a characteristic of the measurement procedure that is not quantifiable (Kacker et al. 2007), for it is a qualitative concept (BIPM 2008). In such instances, the reference should not be done to its accuracy. Instead, it should be done to its uncertainty [usually, the standard uncertainty, which is expressed as the standard deviation of the measurement concerned (BIPM 2008)]. Following the recommendations of the *Comité International des Poids et Mesures* (CIPM), we will use the uncertainty of a measurement to numerically represent its quality (BIPM 2008).

To avoid the limitations of an accuracy and to predict the quality of an indirect measurement from the uncertainty of its input data alone, we define the uncertainty of a measurement as its maximum possible error (*maper*, /maepər/). If a quantity *q* is directly measured as $q_{0}$, then the uncertainty of this measurement in terms of the *maper* is denoted by $\delta$, where $\delta =\max _{q\in S} \left| q-q_{0}\right|$, *S* being the set of all possible values for *q*. For indirect measurements, this is equivalent to the maximum input data error propagation possible. In fact, the *maper* in the indirect measurement of a quantity (measurand) can be determined if the *maper* in the measurement of each of its input quantities are known.

Given a quantity (described by a set of one or more output quantities) $\mathbf {Q}=\left( Q_{1}, \;Q_{2}, \ldots ,\;Q_{m}\right)$ that is calculated as a function of the input quantities $p_{1}, \ p_{2}, \ldots ,\ p_{n}$, let us assume that each of these input quantities are respectively measured as $p_{10}, \ p_{20}, \ldots , \ p_{n0}$. Denoting the *maper* in the measurement of each of these input quantities by $\delta _{1}, \ \delta _{2}, \ldots , \ \delta _{n}$, respectively—i.e., $\delta _{i}=\max _{p_{i}\in S_{i}} \left| p_{i}-p_{i0}\right| , 1\le i\le n$;

$\Delta \left( \mathbf {Q}\left( p_{1}, \ p_{2}, \ldots , \ p_{n}\right) \right)$ denotes the *maper* in the measurement of $\mathbf {Q}$ and it is given by the following equation ($m, \ n\ge 1$):

1 $$\begin{aligned} \Delta \left( \mathbf {Q}\left( p_{1}, \ p_{2}, \ldots , \ p_{n}\right) \right) = \max \left\{ \left\| \mathbf {Q}\left( p_{1}, \ p_{2}, \ldots , \ p_{n}\right) -\mathbf {Q}\left( p_{10}, \ p_{20}, \ldots , \ p_{n0}\right) \right\| \right\} , \nonumber \\ \left| p_{1}-p_{10}\right| \le \delta _{1}, \ \left| p_{2}-p_{20}\right| \le \delta _{2}, \ldots , \ \left| p_{n}-p_{n0}\right| \le \delta _{n} \end{aligned}$$

where $\left\| \mathbf {Y}\right\|$ denotes the length of a vector $\mathbf {Y}=(y_{1},\ldots ,y_{n})$, where $n\ge 1$, given by the square root of an inner product and the difference between a vector $\mathbf {X}$ and a vector $\mathbf {Y}$ is denoted by the vector $\mathbf {XY}$. In symbols, $\mathbf {XY}=\mathbf {Y}-\mathbf {X}$ and $\left\| \mathbf {Y}\right\| =\sqrt{\mathbf {Y}.\mathbf {Y}}=\sqrt{y_{1}^2+y_{2}^2+\cdots +y_{n}^2}$.

This way, Eq. (1) can be a tool to evaluate the quality of the measurement of any quantity, provided that the *maper* of direct measurements can be determined. In particular, the usefulness of this *maper* approach will be shown in this article through its applications to the problem of locating persons using wireless positioning techniques. We will deduce formulae for the *maper* when the coordinates of a person are measured with RFID devices by solving Eq. (1) analytically and deterministically—other approaches to solve the optimization problem given by Eq. (1) are not considered in this article as our solution will show useful in the determination of closed formulae for the optimal ranges for RFID devices, see "Defining and calculating optimal ranges". Since this is an indirect measurement, our formulae are a law of error propagation.

### Defining the problem

**Fig. 2:**
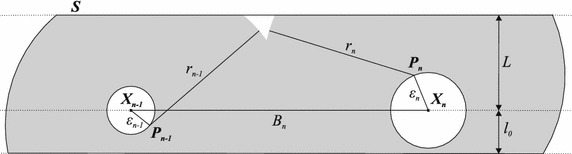
Features related to the problem. In this* figure*, lengths and vectors are presented, in which vectors are* bold*. The region under the coverage of the RFID tags signal is* shaded* and comprises a* union of circles*, each centered on the true position $\mathbf {P_{n}}$ of the *n*th RFID tag and with radius $r_{n}$, $1\le n\le N$. The greatest distance from the corridor’s borders to the line passing through the estimated positions of the tags $\mathbf {X_{n-1}X_{n}}$ is denoted by *L* and the smallest distance by $l_{0}$. The* white circle* centered in the estimated position $\mathbf {X_{n}}$ of the *n*th tag shows the uncertainty of the tag’s position and has a radius $\varepsilon _{n}$, $1\le n\le N$.

We define the problem as follows: given a plane corridor *S* with $L+l_{0}$ width and infinite length; given *N* static RFID tags, $N\ge 1$, attached to points with true positions $\mathbf {P_{1}}$, $\mathbf {P_{2}}, \ldots , \mathbf {P_{n}}, \ldots , \mathbf {P_{N}}$ and with previously measured positions $\mathbf {X_{1}}$, $\mathbf {X_{2}}, \ldots , \mathbf {X_{n}}, \ldots , \mathbf {X_{N}}$ where $\varepsilon _{n}$ is the uncertainty (i.e., the *maper*) corresponding to the measurement of the coordinates of the RFID tag attached to the point $\mathbf {P_{n}}$, $R_{n}$ is the range of the *n*th RFID tag and $h_{n}$ is the height of the *n*th RFID tag above the plane where the user is; given that the user carries an RFID reader, that he is within the region *S*, that he had detected at least one RFID tag, that he is between two RFID tags, that his true position at a given time is $\mathbf {X}$ and that his estimated position by the RFID-based approach at the same given time is $\mathbf {X_{0}}$, the problem is to calculate the *maper* of the user’s position estimation with a wireless positioning technique, where the *maper*, in the light of Eq. (1), is given by Eq. (2); $B_{n}=\left\| \mathbf {X_{n-1}X_{n}}\right\| , \ 1 < n \le N$, $r_{n}=\sqrt{R_{n}^2-h_{n}^2}$, *L* and $l_{0}$ are geometric parameters involved in this problem and illustrated in Fig. 2.

2 $$\begin{aligned} \Delta (\mathbf {X})=\max _{\mathbf {X} \in S} \left\| \mathbf {X_{0}X}\right\| \end{aligned}$$

The *maper* varies according to various factors, including the RFID tags positions uncertainties and the RFID tags positions theirselves. Since the RFID tags are likely to be close to one another, the effect of the uncertainties in the RFID tags positions over the computed user’s position cannot be neglected. At the same time, the preliminary calculus of each RFID tag position is very time-consuming and, depending on the quality standards to be met, may increase costs as their number is likely to be very large. Thus, we consider the RFID tags positions uncertainties into our model to determine the quality that will be required in the measurement of each RFID tag position, in order to meet a quality standard established beforehand.

The solution we will present in the next sections considers the following additional assumptions: the RFID tags have a constant and therefore spherical range $R_{n}$ (this spherical range model will be useful to determine the *maper* when range variations are also taken into account); $\min _{1 \le n \le N}\left\{ r_{n}=\sqrt{R_{n}^2-h_{n}^2}\right\}$ is assumed to be greater than $L+\varepsilon$, where $\varepsilon =\max _{1 \le n \le N}\left\{ \varepsilon _{n}\right\}$. This way, the RFID tags signal covers the whole width of the plane corridor *S* (otherwise, a person walking close enough to one of the corridor’s borders would cross the closest tag without detecting it). Without loss of generality: we assume $\mathbf {X_{n-1}X_{n}}, \ 1 < n \le N$, is parallel to the axis of the corridor; we assume the RFID tags are continuously transmitting their signals (i.e., their latencies are small enough to be neglected); we substitute each $\varepsilon _{n}, \ 1 \le n \le N$, for $\varepsilon =\max _{1 \le n \le N}\left\{ \varepsilon _{n}\right\}$; we assume $\varepsilon <\min _{1 \le n \le N} \left\{ \frac{B_{n}}{2}\right\}$ to preserve the RFID tags topology over *S* and instead of considering each $B_{n}, \ 1 \le n \le N$, each $h_{n}, \ 1\le n\le N$, and each $R_{n}, \ 1\le n \le N$, in particular, we substitute each $B_{n}$ for $B=\max _{1 \le n \le N}\left\{ B_{n}\right\}$, each $h_{n}$ for $h=\max _{1 \le n \le N}\left\{ h_{n}\right\}$ and we consider that the RFID tags have the same range *R*, i.e., $R_{n}=R, \ \forall \ n, \ 1\le n \le N$.

### Exactly modeling the error

To construct the error model we will first compute the *maper* when Cell-Id (Cid) technique is used to locate the user—this technique is also referred to as proximity sensing when RFID devices are considered (Kupper 2005). Cid is the simplest (Bensky 2008; Groves 2008) wireless positioning technique, where the coordinates of the user are the ones of the last (hereafter also referred to as the *n*th, $n\ge 1$) RFID tag read by the user’s RFID reader.

The current error model for Cid technique is to approximate its uncertainty by the size of the cell, or, equivalently, the tag’s maximum range (Kupper 2005; Fu and Retscher 2009; Retscher and Fu 2009). This model is very limited as it does not take into account the uncertainties of the tags positions measurements. Furthermore, the behavior of the uncertainty with respect to the tags range can be quite different, even if, as an extreme case, $\varepsilon$—the uncertainty of the measurement of the tags positions—is neglected. It can be directly verified from the formulae presented in this section that the distance between consecutive tags also play a role on Cid’s *maper*.

**Fig. 3:**
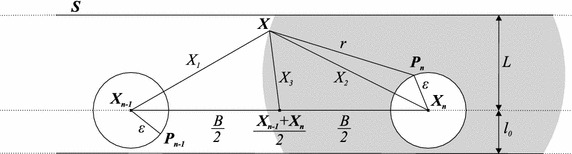
Error modeling of cell-id and mean cell-id techniques. This* figure* illustrates a hypothetical instant when the *maper* is observed. Lengths are typed with* normal font* and vectors with* bold*. The region under the coverage of the *n*th RFID tag’s signal, $1\le n\le N$, is* shaded* and comprises a circle centered on its true position $\mathbf {P_{n}}$ and radius *r*. The greatest distance from the corridor’s borders to the line passing through the estimated positions of the tags $\mathbf {X_{n-1}X_{n}}$ is denoted by *L* and the smallest distance by $l_{0}$. The* white circle* centered in the estimated position $\mathbf {X_{n}}$ of the *n*th tag, $1\le n\le N$, shows the uncertainty of the tag’s position and has a radius $\varepsilon$ for all tags. Depending on the technique employed, the *maper* at the very situation illustrated might be $X_{1}$, $X_{2}$ or $X_{3}$.

In the light of the *maper* concept, the uncertainty of Cid technique is illustrated in Fig. 3 and given by:

3 $$\begin{aligned} \Delta _{Cid}=\max _{\mathbf {X}\in S} \left\{ {\bar{X}}_{1};{\bar{X}}_{2}\right\} \end{aligned}$$

where ${\bar{X}}_{1}$ and ${\bar{X}}_{2}$ are the least upper bounds of $X_{1}=\left\| \mathbf {X_{n-1}X}\right\|$ and $X_{2}=\left\| \mathbf {X_{n}X}\right\|$, respectively.

**Fig. 4:**
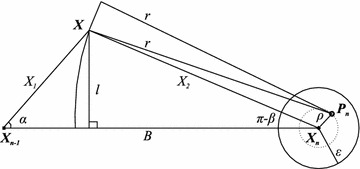
Derivation of the *maper* formulae for cell-id technique. Angles are represented by* greek letters*, vectors by* bold latin letters* and lengths by both* latin* and* greek letters*. The angles are oriented towards an axis parallel to the vector $\mathbf {X_{n-1}X_{n}}$ in a* counterclockwise* direction. The *maper* occurs at the boundary of the region delimited by the tag’s signal, here a circle centered at $\mathbf {P_n}$ and with radius *r*.

To achieve an analytic formula for $\Delta _{Cid}$ we used a number of inequalities. Taking the triangle inequality as starting point, ${\bar{X}}_{1}$ and ${\bar{X}}_{2}$ were both calculated, according to a number of mathematical computations detailed in “Appendix 2” and illustrated in Fig. 4, as (the triangle inequality is applied to the triangle defined by the points $X_{n}$, $P_{n}$ and *X*, Fig. 4):

4 $$\begin{aligned} \left\{ \begin{array}{l} {\bar{X}}_{1}=\left\{ \begin{array}{l} {\bar{X}}_{1a}, \ \text{ if } \ L+\varepsilon \le r \le r_{B} \\ {\bar{X}}_{1b}, \ \text{ if } \ r \ge r_{B} \end{array}\right. \\ {\bar{X}}_{2}=r+\varepsilon , \ r \ge L+\varepsilon \end{array}\right. \ \text{ where: } \left\{ \begin{array}{l} {\bar{X}}_{1a}=\sqrt{B^2+(r-\varepsilon )^2-2B\sqrt{(r-\varepsilon )^2-L^2}} \\ {\bar{X}}_{1b}=\sqrt{B^2+(r+\varepsilon )^2-2B\sqrt{(r+\varepsilon )^2-L^2}} \\ r_{B}=\sqrt{\frac{B^2+L^2-\varepsilon ^2}{B^2-\varepsilon ^2}}B \end{array}\right. \end{aligned}$$

This already solves the problem. But, to obtain direct formulae for $\Delta _{Cid}$ and to derive an optimal range from them, we performed further mathematical work and the direct expression for the *maper* of Cid technique was reached, which is given by Eqs. (5, 6). The deduction of these equations is also in “Appendix 2”.

5 $$\begin{aligned} &\varepsilon < \frac{\sqrt{B^2+L^2}-L}{2} \Rightarrow \nonumber &\quad\Delta _{Cid}\left( R\right) = \left\{ \begin{array}{l} {\bar{X}}_{1a}, \quad \text{ if } L+\varepsilon \le r \le \sqrt{\frac{(B/2)^2+L^2-\varepsilon ^2}{(B/2)^2-\varepsilon ^2}}\frac{B}{2}\\ {\bar{X}}_{2}, \quad \text{ if } \ \ r \ge \sqrt{\frac{(B/2)^2+L^2-\varepsilon ^2}{(B/2)^2-\varepsilon ^2}}\frac{B}{2} \end{array}\right. \end{aligned}$$

6 $$\begin{aligned} \frac{B}{2} > \varepsilon \ge \frac{\sqrt{B^2+L^2}-L}{2} \Rightarrow \Delta _{Cid}\left( R\right) ={\bar{X}}_{2}, \quad \text{ if } \ \ r\ge L+\varepsilon \end{aligned}$$

where ${\bar{X}}_{1a}$ and ${\bar{X}}_{2}$ are given by Eq. (4), $r=\sqrt{R^2-h^2}$

.

We note that though the uncertainties of the tags coordinates are usually not taken into account this is not the case for robotics. There (Meiller and Fabiani 1999), the uncertainty of the position is denoted by u and it also corresponds to the maper. However, the step forward of defining an expression of uncertainty for the maper as done here is not done there.

### Modifying cell-id technique

We propose a modified cell-id technique (MCid, which stands for mean cell-id) as follows: if the RFID reader is detecting two or more RFID tags at a given time, the user’s position is the arithmetic mean of the positions of the last two (i.e., the $n-1$th and the *n*th, $n\ge 2$) RFID tags read; the user’s position is the one of the last (i.e., the *n*th) RFID tag read otherwise. This wireless positioning technique is as simple to implement as cell-id, but, as it will be shown in this article, outperforms it.

**Fig. 5:**
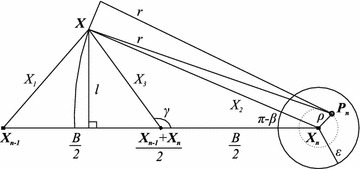
Derivation of the *maper* formulae for mean cell-id technique. Angles are represented by* greek letters*, vectors by* bold latin letters* and lengths by both* latin* and* greek letters*. The angles are oriented towards an axis parallel to the vector $\mathbf {X_{n-1}X_{n}}$ in a* counterclockwise* direction. The *maper* occurs at the boundary of the region delimited by the tag’s signal, here a circle centered at $\mathbf {P_n}$ and with radius *r*—note that now this region may correspond to the intersection of the regions delimited by the last two tags detected, in case at least two tags may be simultaneously detected.

The *maper* for MCid is ruled by the number of tags detected at a given time. This depends on the interval of range considered. If $r<\sqrt{(B/2)^2+L^2}-\varepsilon$ then at most one tag is detected at all times, and hence, the *maper* for MCid is identical to the *maper* for Cid in this case. If $\sqrt{(B/2)^2+L^2}-\varepsilon \le r < \min \left\{ \sqrt{(B/2)^2+L^2}+\varepsilon ;\sqrt{B^2+L^2}-\varepsilon \right\}$ then at most two tags may be detected at a given time. Furthermore, the reader will not be always detecting two tags. Thus, the *maper* is equal to $\max _{\mathbf {X} \in S}\left\{ {\bar{X}}_{1};{\bar{X}}_{2};{\bar{X}}_{3}\right\}$ in this case, where ${\bar{X}}_{1}$, ${\bar{X}}_{2}$ and ${\bar{X}}_{3}$ are the least upper bounds of $X_{1}=\left\| \mathbf {X_{n-1}X}\right\|$, $X_{2}=\left\| \mathbf {X_{n}X}\right\|$ and $X_{3}=\left\| \mathbf {X}-\left( \mathbf {X_{n-1}}+\mathbf {X_{n}}\right) /2\right\|$, respectively, each illustrated by Fig. 3. But, in Eq. (65), "Appendix 3", we show that ${\bar{X}}_{2}$ is greater than ${\bar{X}}_{3}$ in this interval of ranges, thus implying a *maper* equal to $\max _{\mathbf {X} \in S}\left\{ {\bar{X}}_{1};{\bar{X}}_{2}\right\}$ in this same interval of ranges. If $\sqrt{(B/2)^2+L^2}+\varepsilon \le r < \sqrt{B^2+L^2}-\varepsilon$ then one tag is detected at any given time and it is still not possible for the reader to be always detecting two tags. Two tags are detected only at some of the points between the last two tags detected and not at each one of them. This implies a *maper* equal to $\max _{\mathbf {X} \in S}\left\{ {\bar{X}}_{1};{\bar{X}}_{3}\right\}$ in this case. If $r \ge \sqrt{B^2+L^2}-\varepsilon$ then at least one tag is detected at any given time and it may occur, depending on the geometry of the tags, that two tags are always detected, which implies a *maper* equal to $\max _{\mathbf {X} \in S}\left\{ {\bar{X}}_{1};{\bar{X}}_{3};{\bar{X}}_{4}\right\}$ for these ranges. ${\bar{X}}_{4}$ is the least upper bound of $X_{4}$, where $X_{4}=\left\| \mathbf {X}-\left( \mathbf {X_{n-2}}+\mathbf {X_{n-1}}\right) /2\right\|$. But, as we show in Eq. (85), "Appendix 3", $\max _{\mathbf {X} \in S}\left\{ {\bar{X}}_{1};{\bar{X}}_{3};{\bar{X}}_{4}\right\} =\max _{\mathbf {X} \in S}\left\{ {\bar{X}}_{3};{\bar{X}}_{4}\right\}$. Therefore, the uncertainty in the light of the *maper* concept for MCid technique is illustrated in Fig. 3 and given by:

7 $$\begin{aligned} \Delta _{MCid}(R)= \left\{ \begin{array}{l} \max \left\{ {\bar{X}}_{1};{\bar{X}}_{2}\right\} , \quad\text{ if } \ L+\varepsilon \le r < \min \left\{ \sqrt{B^2+L^2}-\varepsilon ;\sqrt{(B/2)^2+L^2}+\varepsilon \right\} \\ \max \left\{ {\bar{X}}_{1};{\bar{X}}_{3}\right\} , \quad \text{ if } \ \sqrt{(B/2)^2+L^2}+\varepsilon \le r < \sqrt{B^2+L^2}-\varepsilon \\ \max \left\{ {\bar{X}}_{3};{\bar{X}}_{4}\right\} , \quad \text{ if } \ r \ge \sqrt{B^2+L^2}-\varepsilon \end{array}\right. \end{aligned}$$

where ${\bar{X}}_{1}$ and ${\bar{X}}_{2}$ are given by Eq. (4), ${\bar{X}}_{3}$ and ${\bar{X}}_{4}$ are given by Eq. (8). The details of ${\bar{X}}_{3}$ and ${\bar{X}}_{4}$ computation are in “Appendix 3”, Fig. 5.

8 $$\begin{aligned}&\left\{ \begin{array}{l} {\bar{X}}_{3}=\sqrt{(B/2)^2+(r+\varepsilon )^2-B\sqrt{(r+\varepsilon )^2-L^2}}, \quad \text{ if } \ r \ge \sqrt{(B/2)^2+L^2}-\varepsilon \\ {\bar{X}}_{4}=\left\{ \begin{array}{l} {\bar{X}}_{4a}, \quad \text{ if } \ L+\varepsilon \le r \le r_{3B/2} \\ {\bar{X}}_{4b}, \quad \text{ if } \ r \ge r_{3B/2} \end{array}\right. \\ \end{array}\right. \nonumber \\&\qquad \qquad \text{ where: } \nonumber \\&\qquad \qquad \left\{ \begin{array}{l} {\bar{X}}_{4a}=\sqrt{(3B/2)^2+(r-\varepsilon )^2-3B\sqrt{(r-\varepsilon )^2-L^2}} \\ {\bar{X}}_{4b}=\sqrt{(3B/2)^2+(r+\varepsilon )^2-3B\sqrt{(r+\varepsilon )^2-L^2}} \\ r_{3B/2}=\sqrt{\frac{(3B/2)^2+L^2-\varepsilon ^2}{(3B/2)^2-\varepsilon ^2}}\frac{3B}{2} \end{array} \right. \end{aligned}$$

The direct formulae to the problem are given by Eqs. (9–11). The deductions are also detailed in “Appendix 3”.

9 $$\begin{aligned}&\varepsilon \le \frac{\sqrt{B^2+L^2}-\sqrt{(B/2)^2+L^2}}{2} \Rightarrow \nonumber \\&\qquad \Delta _{MCid}\left( R\right) = \left\{ \begin{array}{ll} {\bar{X}}_{1a}, & \text{ if } \ \ L+\varepsilon \le r< \sqrt{\frac{(B/2)^2+L^2-\varepsilon ^2}{(B/2)^2-\varepsilon ^2}}\frac{B}{2} \\ {\bar{X}}_{2}, & \text{ if } \ \ \sqrt{\frac{(B/2)^2+L^2-\varepsilon ^2}{(B/2)^2-\varepsilon ^2}}\frac{B}{2} \le r <\sqrt{\left( B/2\right) ^2+L^2}+\varepsilon \\ {\bar{X}}_{1a}, & \text{ if } \ \ \sqrt{\left( B/2\right) ^2+L^2}+\varepsilon \le r < \sqrt{\frac{(3B/4)^2+L^2-\varepsilon ^2}{(3B/4)^2-\varepsilon ^2}}\frac{3B}{4} \\ {\bar{X}}_{3}, & \text{ if } \ \ \sqrt{\frac{(3B/4)^2+L^2-\varepsilon ^2}{(3B/4)^2-\varepsilon ^2}}\frac{3B}{4} \le r < \sqrt{B^2+L^2}-\varepsilon \\ {\bar{X}}_{4a}, & \text{ if } \ \ \sqrt{B^2+L^2}-\varepsilon \le r < \sqrt{\frac{B^2+L^2-\varepsilon ^2}{B^2-\varepsilon ^2}}B \\ {\bar{X}}_{3}, & \text{ if } \ \ r \ge \sqrt{\frac{B^2+L^2-\varepsilon ^2}{B^2-\varepsilon ^2}}B \end{array}\right. \end{aligned}$$

10 $$\begin{aligned}&\frac{\sqrt{B^2+L^2}-L}{2} > \varepsilon > \frac{\sqrt{B^2+L^2}-\sqrt{(B/2)^2+L^2}}{2} \Rightarrow \nonumber \\&\qquad \Delta _{MCid}\left( R\right) = \left\{ \begin{array}{ll} {\bar{X}}_{1a}, & \text{ if } \ \ L+\varepsilon \le r< \sqrt{\frac{(B/2)^2+L^2-\varepsilon ^2}{(B/2)^2-\varepsilon ^2}}\frac{B}{2} \\ {\bar{X}}_{2}, & \text{ if } \ \sqrt{\frac{(B/2)^2+L^2-\varepsilon ^2}{(B/2)^2-\varepsilon ^2}}\frac{B}{2} \le r < \sqrt{B^2+L^2}-\varepsilon \\ {\bar{X}}_{4a}, & \text{ if } \ \ \sqrt{B^2+L^2}-\varepsilon \le r < \sqrt{\frac{B^2+L^2-\varepsilon ^2}{B^2-\varepsilon ^2}}B \\ {\bar{X}}_{3}, & \text{ if } \ \ r \ge \sqrt{\frac{B^2+L^2-\varepsilon ^2}{B^2-\varepsilon ^2}}B \end{array}\right. \end{aligned}$$

11 $$\begin{aligned} \min \left\{ \frac{B}{2};\frac{\sqrt{4B^2+L^2}-L}{2}\right\} > \varepsilon \ge \frac{\sqrt{B^2+L^2}-L}{2} \Rightarrow \nonumber \\ \Delta _{MCid}\left( R\right) = \left\{ \begin{array}{ll} {\bar{X}}_{4a}, &\text{ if } \ \ L+\varepsilon \le r < \sqrt{\frac{B^2+L^2-\varepsilon ^2}{B^2-\varepsilon ^2}}B \\ {\bar{X}}_{3}, & \text{ if } \ \ r \ge \sqrt{\frac{B^2+L^2-\varepsilon ^2}{B^2-\varepsilon ^2}}B \end{array}\right. \end{aligned}$$

12 $$\begin{aligned} \frac{B}{2} > \varepsilon > \frac{\sqrt{4B^2+L^2}-L}{2} \Rightarrow \Delta _{MCid}(R)= {\bar{X}}_{3}, \ \text{ if } \ r\ge L+\varepsilon \end{aligned}$$

where ${\bar{X}}_{1a}$ and ${\bar{X}}_{2}$ are given by Eq. (4), ${\bar{X}}_{3}$ and ${\bar{X}}_{4a}$ are given by Eq. (8), $r=\sqrt{R^2-h^2}$.

### Defining and calculating optimal ranges

For broad is the spectrum of possible ranges for the RFID tags (Glover and Batt 2006) used in this location technology we propose an optimal range (*orange*). This way, time and costs could be reduced because the formulae would take the place of the trials and faster, better assessed choices could be made when the tags are about to be purchased or even manufactured as its spectrum of possible ranges is dramatically narrowed.

Since we have deduced a set of analytic and direct formulae for the *maper* of each presented technique, the *orange* is defined as the one for which the *maper* is minimal. By doing so, the quality of an RFID-based location approach is expected to be maximized.

First, if the set of possible ranges is finite, such as a finite set of pre-defined values established by manufacturers or offered by sellers, then the *orange* is obtained by substituting each of the range candidates in the *maper* formulae and checking for which one the *maper* is the least. Otherwise, if there are infinite possibilities, then the *orange* can be obtained by studying the *maper* as a function of the range. After some mathematical work, outlined in “Appendix 4”, the *oranges* of both Cid and MCid techniques considering the entire spectrum of ranges, $R_{Cid}$ and $R_{MCid}$ respectively, are obtained. For Cid technique, the *orange* follows:

13 $$\begin{aligned} R_{Cid}=\sqrt{r_{Cid}^2+h^2} \end{aligned}$$

where $r_{Cid}$ is given by Eqs. (14) and (15).

14 $$\begin{aligned} \varepsilon < \frac{\sqrt{B^2+L^2}-L}{2} \Rightarrow r_{Cid}=\sqrt{\frac{(B/2)^2+L^2-\varepsilon ^2}{(B/2)^2-\varepsilon ^2}}\frac{B}{2} \end{aligned}$$

15 $$\begin{aligned} \frac{B}{2} > \varepsilon \ge \frac{\sqrt{B^2+L^2}-L}{2} \Rightarrow r_{Cid}=L+\varepsilon \end{aligned}$$

The *orange* of MCid technique is given by:

16 $$\begin{aligned} R_{MCid}=\sqrt{r_{MCid}^2+h^2} \end{aligned}$$

where $r_{MCid}$ is given by Eqs. (17–19).

17 $$\begin{aligned} \varepsilon \le \frac{\sqrt{B^2+L^2}-\sqrt{(B/2)^2+L^2}}{2} \Rightarrow r_{MCid}=\sqrt{\frac{(3B/4)^2+L^2-\varepsilon ^2}{(3B/4)^2-\varepsilon ^2}}\frac{3B}{4} \end{aligned}$$

18 $$\begin{aligned} \min \left\{ \frac{\sqrt{4B^2+L^2}-L}{2};\frac{B}{2}\right\} > \varepsilon > \frac{\sqrt{B^2+L^2}-\sqrt{(B/2)^2+L^2}}{2} \Rightarrow r_{MCid}=\sqrt{\frac{B^2+L^2-\varepsilon ^2}{B^2-\varepsilon ^2}}B \end{aligned}$$

19 $$\begin{aligned} \frac{B}{2} > \varepsilon \ge \frac{\sqrt{4B^2+L^2}-L}{2} \Rightarrow r_{MCid}=L+\varepsilon \end{aligned}$$

For reference, we are providing an ODS spreadsheet with the maper and *orange* formulae as a shared link available at mendeley.com/profiles/eduardo-del-rio, see Del Rio (2011). There, their outputs can be studied for any desired instance as its cells may be freely edited by everyone. We included both the direct and indirect maper formulae of each technique in it.

### Addressing range variations

For any solid there are always two solids that interact with it in the following way: the one contains it and the other is contained by it. Further, these two solids can always be taken for spheres. This obvious mathematical fact is laid as the foundation to model the error when varying ranges, as opposed to the constant ones assumed thus far, are considered (it is implicit that the solids dealt with in this paper have no holes).

**Fig. 6:**
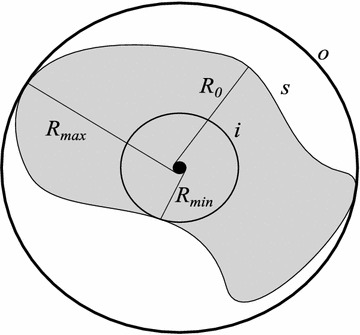
Varying ranges and the two-spheres approximation.* Aerial view* of the region covered by a hypothetical tag (*grayed region*) located at the figure’s center (*black dot*). This region’s section is denoted by *s* and has an average radius $R_{0}$. The inner and the outer circles of the two-spheres approximation are denoted by *i* and *o* respectively. Their radii $R_{min}$ and $R_{max}$ correspond to the minimum and maximum distance from the tag’s range surface to the tag itself.

To address the computation of the *maper* when a generic surface, rather than spherical, is considered, we will approximate the solid corresponding to the region covered by the RFID tags signal based on two concentric spheres of revolution, the one to contain it and the other to be contained by it, Fig. 6. These spheres are generated by rotating two circles among an axis containing any of their own, respective, diameters. Regarding these circles, they are concentric; both centered at the true position of a tag. The first circle (namely the inner circle) contains the plane region defined by the intersection between the generic range surface and the plane corridor *S* where the user is. Among all these, it corresponds to the one with the least radius. The second circle (namely the outer circle) is inside the plane region defined by the intersection between the generic range surface and the plane corridor *S* where the user is. Among all these, it corresponds to the one with the greatest radius.

Two possibilities are presented to compute the *maper* when varying ranges are taken into account in the light of the two-spheres approximation just described. They set an upper bound for the *maper* based on the *maper* formulae for spherical range surfaces heretofore presented. The first approach is to bound the *maper* by $\tilde{\Delta }(R_{0},K)$, according to the following equation:

20 $$\begin{aligned} \tilde{\Delta }(R_{0},K)=\max _{\left| R-R_{0}\right| \le K}\left\{ \Delta (R)\right\} , \ R_{0} \ge K+\sqrt{(L+\varepsilon )^2+h^2} \end{aligned}$$

where $R_{0}$ is the nominal range, *K* is the *maper* of the range’s measurement—or equivalently, the maximum possible magnitude of range variations—and $\Delta (R)$ is the *maper* of the technique in consideration in the absence of range variations. As a condition, $R_{0}-K\ge \sqrt{(L+\varepsilon )^2+h^2}$ must be satisfied; otherwise, $\tilde{\Delta }(R_{0},K)$ will not be defined. If the tags have a nominal range—e.g. as advertised by their seller on the time of their purchase or as a choice to be done prior to their production or, still, an average range—but, in practice, it is observed that the signal is transmitted over different distances then *K* can be equaled to the maximum variation in magnitude from the nominal range. In other words, the tags have a range $R_{0}$ which may vary from $R_{0}-K$ to $R_{0}+K$, the radii of the inner and the outer circle respectively. Moreover, the *maper*$\Delta (R_{0},K)$ relates to its approximation $\tilde{\Delta }(R_{0},K)$ as follows:

21 $$\begin{aligned} \tilde{\Delta }(R_{0},K) \ge \Delta (R_{0},K), \ \forall K\ge 0, R_{0} \ge K+\sqrt{(L+\varepsilon )^2+h^2} \end{aligned}$$

The *maper* can also be approximated as $\tilde{\Delta }(R_{min},R_{max})$, which is given by the following equation:

22 $$\begin{aligned} \tilde{\Delta }(R_{min},R_{max})=\max _{R_{min}\le R\le R_{max}}\left\{ \Delta (R)\right\} , \quad R_{min} \ge \sqrt{(L+\varepsilon )^2+h^2} \end{aligned}$$

where $R_{max}$ and $R_{min}$ are the maximum and the minimum range observed in practice, respectively. As a condition, $R_{min}$ must satisfy $R_{min}\ge \sqrt{(L+\varepsilon )^2+h^2}$. For the numerical tests performed in the current article, only Eq. (20) was considered. It should be noted that Eqs. (20) and (22) are identical since Eq. (22) is derived from Eq. (20) by letting $R_{0}=\left( R_{max}+R_{min}\right) /2$ and $K=\left( R_{max}-R_{min}\right) /2$ in it. Moreover, the actual *maper*$\Delta (R_{min},R_{max})$ is related to its approximation $\tilde{\Delta }(R_{min},R_{max})$ as follows:

23 $$\begin{aligned} \tilde{\Delta }(R_{min},R_{max}) \ge \Delta (R_{min},R_{max}), \ \forall R_{min} \ge \sqrt{(L+\varepsilon )^2+h^2} \end{aligned}$$

In like manner, the *orange* can be redefined if Eq. (20) or (22) is considered to estimate the *maper*. For example, if Eq. (20) is considered, the *orange* would be the $R_{0}$ for which: $\tilde{\Delta }(R_{0},K)$ is defined; $\tilde{\Delta }(R_{0},K)$ is minimized. In addition, instead of an analytic computation of the *orange* formula for this situation, its value may be computed numerically.

Thus, Eqs. (20) and (22) also addressed issues like variation in tag and reader antenna sensitivity with direction; reflection of signals off floor, ceilings and walls that will produce both constructive and destructive interference; variation in read sensitivity between different models; variation in tag performance with aging; variation in range due to body shadowing; since each one of these issues will produce, in the light of Cid and MCid techniques, the same effect: variations on the RFID tags ranges. In other words, variations from the spherical range surface model to a generic range surface model.

### Locating emergency calls in real-time

Safety of life is a public concern all over the world. In particular, the US is concerned about the localization of emergency phone calls in real time, in the sense that they are establishing quality standards (Bensky 2008) for the localization of mobile radio services. A new emergency calls service, named Enhanced 911 service (E911), in its phase II, obligated every handset based technologies to provide by September 11, 2012 the location of an emergency phone call with the uncertainties (FCC 2010): 50 m for 67% of the phone calls; 150 m for 95% of the phone calls. GPS is likely to be out of service indoors. In regard to urban regions, it may have an uncertainty worst than E911 standards (Do et al. 2007; Prost 2008) as well, depending on the particular urban environment where the localization takes place.

#### State-of-the-art of location technologies

The effort to meet FCC requirements and also to provide other Location Based Services (LBS) gave rise to a variety of urban and indoor positioning solutions. Some alternatives to GPS are: the cellular towers identification, which is the simplest and, at the same time, the most degraded approach, providing (Bensky 2008; Groves 2008) an uncertainty within kilometers; the cellular trilateration, which provides (Bensky 2008; Groves 2008) an uncertainty within hundreds of meters. Examples of recent and more accurate urban and indoor positioning solutions are based on the integration of GPS with other location technology like cellular (De Lorenzo 2009) and TV (Do et al. 2007) trilateration. Among the approaches presented, the ones that meet E911 standards are based on GPS. However, GPS technology is usually not available indoors and it may well perform poorly in urban regions.

**Table 1:** *Maper* and *oranges* for Cid technique

$\varepsilon$	*B*	$R_{Cid}$	$\Delta _{Cid}\left( R_{Cid}\right)$	$\tilde{\Delta }_{Cid}\left( R_{Cid},10\right)$	$\tilde{\Delta }_{Cid}\left( R_{Cid},20\right)$
2	35	29.2	28.7	#	#
5	35	29.8	32.2	#	#
10	35	32.3	40.0	#	#
2	70	42.1	42.3	54.6	#
5	70	42.2	45.4	58.8	#
10	70	42.5	50.7	70.2	#

*Maper* and *Oranges* of Cell-Id Technique for $B=35\;m$ and $B=70\;m$, where *B* is the distance between consecutive RFID tags and maximum variations of $K=10, \ 20\;m$ are admitted on the tags ranges magnitude in the light of Eq. (1). The results are in meters and $h=12\;m$, $L=20\;m$, $l_{0}=5\;m$. Fields marked with a # are those where there is at least a range $r=\sqrt{R^2-h^2}$ for which $r\ge L+\varepsilon$ is not satisfied.

Since RFID tags are replacing bar codes (Peterson 2001; Glover and Batt 2006) as its prices fall and taking into consideration technical difficulties and cost to deploy network-based solutions (Catuto et al. 2010), we seek a handset-based solution to urban and indoor positioning based solely on RFID devices. In regard to it, pedestrians had been located over small urban areas of Vienna, Austria, in real time using only Cid technique (Fu and Retscher 2009). The obtained uncertainty for the singular urban environment considered was about 20 m (Fu and Retscher 2009), which satisfies FCC standards for their E911. Inside indoor areas, if RSSI data is used uncertainties within few meters may be achieved (Fu and Retscher 2009; Retscher and Fu 2009).

#### Limitations of RFID-handset-based approaches

To date, the way in which the tags are to be distributed over urban and indoor regions was not yet taken into account or discussed. Moreover, despite good uncertainties already achieved (Fu and Retscher 2009; Retscher and Fu 2009), the distribution of the tags becomes an issue when it comes to the implementation of this approach over larger and more complicated environments that will demand much more tags, such as the entire urban regions of a major city, including the indoor areas within them. After all, as active tags were used (Fu and Retscher 2009; Retscher and Fu 2009), which are currently among the most expensive and require batteries to work, some issues arise (noteworthy, currently, passive tags cannot be used for positioning especially outdoors due to their limited range which is the reason why other authors and we have focused on active tags): the need to install each of the tags in the urban and indoor environments prior to the implementation of the location system; the need for periodic replacement of the tags batteries, as they must be always transmitting their signals (here, this does not mean that their latency is zero, but rather, that they must not be turned off), since the goal is to locate emergency calls in real time (this “continuous” transmission decreases the life of the tags batteries); the need for periodic replacement of the tags due to aging; the need to survey each of the tags prior to the implementation of the location system (i.e., calculate the coordinates of each one of them). Due to the large number of tags required for implementation, addressing these issues is time-consuming and increases costs.

Since there are countless ways to convey a distribution of tags, a fundamental question comes to light: how to smartly distribute the tags over urban and indoor environments in order to address as many of these issues employing as few tags as possible and still meet E911 quality standards?

#### A tags-in-lamps approach

To develop cheaper active RFID tags will certainly decrease the overall cost of this approach, but it will not answer this question as it does not address the issues afore posed. Therefore, we propose an approach to distribute RFID tags over urban and indoor areas as follows: in urban areas, RFID tags are attached to the lamps of the lamp-posts on the urban streets, or to the lamp-posts; in indoor areas the approach is similar, except for the fact that the RFID tags are now attached to the lamps of the ceiling or directly to the ceiling. Due to the greater density of RFID tags in the indoor approach—the lamps are closer to each other—its accuracy will be better than that of the urban approach. For reference, the user carries an RFID reader and RSSI readings are not used as input data, even if the RFID reader is able to determine them; Cid or MCid technique is used to estimate the user’s position in real-time.

**Table 2:** *Maper* and *oranges* for MCid technique

$\varepsilon$	*B*	$R_{MCid}$	$\Delta _{MCid}\left( R_{MCid}\right)$	$\tilde{\Delta }_{MCid}\left( R_{MCid},10\right)$	$\tilde{\Delta }_{MCid}\left( R_{MCid},20\right)$
2	35	35.1	23.0	*38.6*	#
5	35	35.3	25.0	#	#
10	35	42.5	35.3	52.8	#
2	70	57.5	28.0	*36.8*	*47.6*
5	70	57.5	30.4	*39.7*	51.0
10	70	57.6	34.6	60.3	60.3

*Maper* and *oranges* of mean cell-id technique for $B=35\;m$ and $B=70\;m$, where *B* is the distance between consecutive RFID tags and maximum variations of $K=10, \ 20\;m$ are admitted on the tags ranges magnitude in the light of Eq. (20). The results are in meters and $h=12\;m$, $L=20\;m$, $l_{0}=5\;m$. Fields marked with a # are those where there is at least a range $r=\sqrt{R^2-h^2}$ for which $r\ge L+\varepsilon$ is not satisfied.

Values that comply with FCC standards (lesser than 50 m) are highlighted in italics.

#### Its implementation

The implementation of this approach takes place in the following steps: establishing the set of lamps to be used (e.g. all the lamps or half of the lamps); surveying the positions of each lamp belonging to this set (e.g. using total stations to compute their coordinates through the measurement of distances and angles as for outdoors and using the buildings blueprints as for indoors); attaching an RFID tag to each lamp (or, alternatively, the lamp-posts post and indoors ceilings) of this set. Now, if the tags are not able to send their own coordinates through their signals but their unique identities alone, then two additional steps are required: to create a database that associates a set of coordinates (e.g. latitude, longitude and height on a reference frame; ITRF2008 if one wishes) to the unique identity of each tag; embed this database into the positioning software of the user’s location system, which is based on the standard Cid or on the MCid technique proposed here and comprises the reader and a processing unit. For instance, the processing unit can be a laptop to which the RFID reader is connected through a serial/RS232 port or a USB port—as for this article, without loss of generality, the user carries the laptop with an RFID reader connected to it, c.f. our own field experiments (Del Rio 2009, 2010).

This way, while the user walks over the streets and indoors his position is simply calculated from the coordinates of the tags (the tags will be transmitting their signals all the time, in which their unique identities are embedded) in his vicinity. His position is estimated by equaling his own coordinates to the ones of the last tag detected or to the arithmetic mean of the ones of the last two tags detected (after reading the tags unique identities, their coordinates will be retrieved from the database constructed and embedded into the processing unit beforehand), regardless of the number of tags being detected at this very time—as far as the user’s RFID reader had detected at least one tag over the course of time. In addition, a periodic replacement of the tags batteries and, probably, of the tags their selves is required, in principle, to cope with the limited life span of the batteries and with the variation in the tags performance as they grow old.

**Table 3:** *Maper* and *oranges* for Cid and MCid technique considering a maximum range

$\varepsilon$	*B*	$R_{max}$	$\tilde{\Delta }_{Cid}\left( R_{min},R_{max}\right)$	$\tilde{\Delta }_{MCid}\left( R_{min},R_{max}\right)$
2	35	65.1	64.0	*47.7*
5	35	67.7	70.0	53.5
10	35	72.3	80.0	63.2
2	70	65.1	72.8	72.8
5	70	67.7	72.8	72.8
10	70	72.3	80.0	72.8

*Maper* of cell-id and mean cell-id technique for $B=35\;m$ and $B=70\;m$, where *B* is the distance between consecutive RFID tags and the amplitude of range variations is $R_{max}-R_{min}=40\;m$, in the light of Eq. (22). The results are in meters and $h=12\;m$, $L=20\;m$, $l_{0}=5\;m$. $r\ge L+\varepsilon$, with $r=\sqrt{R^2-h^2}$, is always satisfied because it corresponds to $r_{min}$.

Values that comply with FCC standards (lesser than 50 m) are highlighted in italics.

#### The formulae

If there are two or more possible ranges for the RFID tags to be employed, then the *maper* and the *orange* formulae presented in the preceding sections can be used to decide which one should be adopted for this positioning system. Our *maper* formulae are also a tool to define the set of lamps to which the tags are to be attached—this will be demonstrated by a numerical experiment described later on in this subsection—and to address the tags surveying. In these formulae, the quality of a surveying technique is represented by $\varepsilon$. From the good or bad quality of a technique follows a lesser or a greater $\varepsilon$, respectively. This way, the *maper* of the user’s position estimation can be known prior to surveying the tags. Thus, the best surveying technique might be chosen based on cost-benefits criteria prior to the surveying itself. After all, it is not mandatory to survey the positions of all tags with the same quality; e.g. tags within less densely populated urban regions could be surveyed with the worst, and likely, cheapest possible technique only for basic applications such as E911.

#### Some grounds

Some grounds for choosing the lamps of the lamp-posts and ceilings as the place for the RFID tags are stated over this paragraph. The lamps are regularly distributed over the urban streets and indoors and the distance between consecutive lamp-posts may have an official value. For example, in the Brazilian state of São Paulo this distance is established by Eletropaulo (Santos 2008)—the energy supplier of São Paulo—as 35 m; the level difference between the RFID tags and the RFID reader of the user decreases the effects of the NLOS. In fact, Eletropaulo (Santos 2008) states for the lamp-posts heights a value between 10 and 12 m and as for indoors, their ceilings are at a higher level than their dwellers; the energy supply of the lamps could also be used by the RFID tags as their main power source, allowing greater ranges, lesser latencies or even a continuous transmission and definitely dismissing the use of batteries; the tag’s installation and periodic replacement could be performed in conjunction with the periodic replacement of the lamps; the prices of RFID devices are diminishing and a mass production to implement our proposed approach could be an adding factor to decrease their cost. Furthermore, compared to GPS, it does not require advanced technologies and it has a simpler implementation, as GPS is based (Bensky 2008; Groves 2008) on a particular satellite constellation and on precise time estimations using atomic clocks. However, our approach is not global like GPS but local, as it must be set on a previously prepared environment.

#### The numerical experiment

To study the performance of our approach, we applied the *maper* concept to two scenarios, both based in São Paulo: in the first scenario, every lamp-post is RFID tagged; in the second scenario, the lamp-posts are alternately tagged with RFID tags, there are not two consecutive lamp-posts with RFID tags attached and there are not three consecutive lamp-posts without RFID tags attached. We assume the distance between consecutive lamp-posts is 35 m as stated by Santos (2008), the urban streets width (including both of its sidewalks) is 25 m, the lamp-posts heights are 12 m and the ranges of the RFID tags used are the optimal ones. Taking into account the dynamics of the urban environment with its great number of vehicles, people, buildings and in some cases even trees (as well as other error sources highlighted in the next subsection) we consider that the range, *R*, of the RFID tags is not constant. Instead, we assume that it may vary by 20 m in magnitude. In other words, the ranges are within the following interval: $R-20\le R\le R+20$—noteworthy, the amplitude of range variations is actually 40 m. Under these hypotheses, the *maper* of Cid and MCid techniques was calculated with our formulae. For instances in which range variations were considered, the corresponding *maper* was computed by Eq. (20), introduced in the preceding section. Now considering that the range is always greater than $L+\varepsilon$, the *maper* is computed considering an amplitude of range variations equals 40 m, i.e. $R_{max}-R_{min}=$ 40 m. This way, the corresponding *maper* can be computed by Eq. (22).

The results are displayed on Tables 1, 2 and 3, respectively. It should be noted that even with 40 m variations in the magnitude of the ranges of the RFID tags used, E911 standards were met with MCid, since a *maper* lesser than 50 m means that the uncertainty is lesser than 50 m at 100% of the time. For most of the situations considered in this numerical experiment, MCid technique provided better accuracies than Cid. Moreover, if E911 standards are to be met in the light of range varations, the formulae tells the quality required in the knowledge of tags’s coordinates. For São Paulo, it should not be worse than 2 m.

## Discussion

In this article we developed an analytic and deterministic error model for the most basic wireless positioning techniques. In the End, the *maper* mapped the error and produced *oranges*. Moreover, the *maper* can be regarded as a parameter to evaluate the quality of measurements in general, since it was generally defined prior to its application to location techniques. This way, the quality metric and the law of error propagation comprised by the *maper* concept might be used in distinct scientific and technological instances as well. For example, although we consider an RFID-based location technology, our formulae can also be applied to a cellular-based technology. In this case, the RFID tags are replaced by cellular tower antennas, the RFID reader is replaced by a cell-phone and the range of the RFID tags would now be the one of the cellular tower antennas. However, the topology of cellular antennas is different from the linear distribution of RFID tags over streets, subways and buildings corridors. Thus, the formulae of this article provide an upper bound for the possible errors of positioning techniques with cellular signals but not the *maper* (i.e., the least upper bound). To enhance the model for a cell-phone based technology and to evaluate the corresponding *maper*, the problem and the MCid technique should be restated for a triangular geometry of cellular antennas. Another point regarding cellular-based location technologies is that the uncertainty of the coordinates of the cellular antennas is very small compared to the distance between neighboring antennas and to the distance between the antennas and the cell-phone of the user. Hence, the error model for a cellular-based technology can be constructed assuming $\varepsilon =0$. Moreover, the formulae we have deduced ensemble an *orange*, which was defined and deduced here.

Field experiments are not presented but rather, only numerical experiments. Although, the positioning in urban and indoor areas based solely on RFID technology is indeed feasible (Fu and Retscher 2009). In fact, the positioning in urban areas can be performed using Cid and even better results are achieved in indoor areas by considering RSSI data (Fu and Retscher 2009; Retscher and Fu 2009). Nevertheless, the fundamental issue of how to distribute the tags over the large and complicated urban and indoor environments remained open. We brought it to light and we proposed a way to address it in this article.

### Outcomes of mean cell-id technique

The drawback of our solution to the problem is the variation on the RFID tags range due to urban and indoor composition and dynamics and other error sources, since we have assumed a spherical and therefore constant range. Nevertheless, our model remains consistent as variable ranges can be inserted into it and the corresponding *maper* computed by either Eqs. (20) or (22). The procedure could be that of the previous section, in which E911 standards were shown to be met with the proposed MCid technique, even when the range varied from the optimal range by 20 m in magnitude in the light of Eq. (20), Table 2. The MCid technique provided better uncertainties than Cid and this improvement was enhanced when range variations took place.

Surprisingly, in the light of range variations from the optimal, the best results were achieved in the second implementation scenario, which uses only half of the tags considered in the first scenario and is therefore, the smartest distribution so far, see Table 2. In the second scenario, time, labor and costs related to purchase, installation, surveying and maintenance of the tags are cut roughly by half and, still, E911 standards are met even if the tags range vary by 20 m in magnitude from the optimal one. What is more, the implementation of our tags-in-lamps approach under this circumstance was only feasible through the MCid technique proposed. We see that even with range variations the use of the optimal range significantly improves the quality of the location system, compare Tables 2 and 3. Without optimal ranges but under the same amplitude of range variations E911 standards could only be met in the first implementation scenario which requires the double of tags, see Table 3 (and again E911 are met only through the MCid technique proposed here).

### Low-cost improvements of the solution

Additionally, RSSI data could be used to deal with range variations. Usually, RFID readers are able not only to detect the signal of nearby tags, but also to determine the strength of the tag’s signal that is received at the time of the reading. This quantity varies according to the distance between the source and the receiver of the signal (Bensky 2008) as it generally decreases when the source–receiver distance is increased. This signal’s power information is usually available as RSSI data (Fu and Retscher 2009; Retscher and Fu 2009). Therefore, the variations of range observed in practice could be also addressed by the incorporation of RSSI data into Cid and MCid techniques. The detection of a tag would be redefined in the light of the RSSI data. For instance, the tags identified by the reader at a certain time and with a RSSI corresponding to a signal’s power below a specified threshold would not be taken for detected tags but neglected. This way, tags detected too far away from the nominal or average range would not interfere with the quality of the location and *K* would be diminished, thus, decreasing the uncertainty achieved [Eqs. (20) and (22) remain valid for this technique as this change in the algorithms translates solely as a decrease in the magnitude of range variations]. Moreover, uncertainties within few meters had been achieved by employing this approach in indoor areas (Retscher and Fu 2009).

Specific applications might require a much higher quality than the one standardized by FCC in their E911. In the light of our RFID approach, improvements are feasible by integrating an inertial navigation system (INS) to it. An INS is based on dead-reckoning (Groves 2008), a location technique that depends on recurrent updates of the location of the user. Thus, an INS would compute an accurate location using the RFID tags positions to make the required periodic updates. In indoor areas, uncertainties about 1.00 m may be achieved through the integration of RFID with a low-cost INS (Retscher and Fu 2009). This way, specific users seeking for an improved solution may, independently and on their own, integrate an INS to their private RFID readers.

### Practical remarks

The establishment of the *maper* as a benchmark was useful since it led to a singular expression of uncertainty and to *oranges*. However, the *maper* for Cid and MCid techniques is unlikely to be perfectly determined in practice because it is a function of $\varepsilon$ and it might also be a function of *K*, parameters that are both defined in terms of the *maper*. In an implementation scenario, an alternative is to consider the standard uncertainty—i.e., standard deviations—of the measurements associated to these parameters by choosing a coverage factor (BIPM 2008). Thus, $\varepsilon$ and *K* can be both equaled to the double of the standard deviation (SD) corresponding to the measurement of the tags positions and to the measurement of their ranges, respectively. This way, if a Gaussian distribution is assumed then the probability that these quantities are within the interval of confidence delimited by the sd increased twofold will be about 95%, which is still close to the 100% achieved with the *maper*. Otherwise, $\varepsilon$ and *K* could be both equaled to 2.5 or even 3 times the SD of each of the corresponding measurements or distinct probability distributions could be tried in order to choose a reasonable coverage factor. Technically, this would not be the *maper*, but an uncertainty corresponding to a highly likely maximum error.

Furthermore, Eqs. (20) and (22) are an upper bound for the *maper*, but not the *maper* (i.e., the least upper bound) itself. Notwithstanding, from the mathematics of its construction and the results achieved in the numerical experiments, the two-spheres approximation given by Eqs. (20) and (22) is sound inasmuch as the 100% probability in attaining the interval of confidence provided is preserved and the *maper* is actually lesser than this approximation, Eqs. (21) and (23).

In the experiments performed it was assumed that the ranges may vary by at most 20 m in magnitude—i.e., $K\le 20\text{ m }$. However, though the true amplitude of such variations is unknown—which means that they can be either much less or much more than that—Eqs. (20) and (22) span any theoretically possible value for these range variations. In other words, though we lack empirical evidence to state the value of *K* or one of its upper bounds, our formulae predict de facto the *maper* and the *orange* (regardless of *K*’s magnitude).

### Towards ubiquitous positioning

The non-dependence on estimations of distances, angles, time intervals and received signal strength indications points up to the ease of implementation, regardless of environment preparations. Our error model provides consistent results with reality in the light of input parameters and previous work on urban and indoor location based solely on RFID. What is more, the RFID-based approach proposed in this article is independent from other location technologies and it might locate emergency calls in real time using the pre-existing lamp-posts and indoor lamps structure. This greatly diminishes the implementation’s labor, which comprises the measurement of each RFID tag coordinates and their individual installation—analogous to the installation of lamps over the streets and indoors. It also simplifies the tags periodic replacement, as it is likely that unified operations to simultaneously replace lamps and RFID tags will be feasible. And there is more, the energy supply of the lamps could serve as the tags main power, dismissing the use of batteries and allowing a continuous transmission of the signals along the great ranges required by this approach.

Last, in a large scale implementation scenario, every single lamp produced could embed an RFID tag (i.e., these lamps would transmit not only light but an RFID signal as well). The range of an embedded tag would be ruled by the type of the respective lamp. This way, their ranges could be chosen based on the *orange* formulation presented in this article and just as easily as to pick oranges. If such “radio-lamps” are feasible, ubiquitous positioning would draw nigh at hand.

## Conclusions

The expression of uncertainty proposed here was succesfully applied to positioning with RFID. Its mathematical formulation as an optimization problem makes it suitable to be used as a law of error propagation. It can be used to measure uncertainties in the absence of direct measurements of the quantity concerned and when the standard uncertainty fails. Moreover, it provides a 100% likely confidence interval. However, it should be pointed out that the concept of the maper is not per se new as it is also considered in robotics (Meiller and Fabiani 1999).

Here, the usefulness of the *maper* was shown by studying the urban and indoor positioning with RFID devices. The *maper* and *orange* formulae were shown here and constitute a means of determining parameters of a RFID location system prior to its implementation. For example, using the proposed formulae two implementation scenarios were studied and several conclusions could be drawn: MCid technique usually provide significantly more certain results than Cid technique; under range variations E911 standards can still be met if MCid technique is used and the tags coordinates are known with a specified quality; MCid is more robust than Cid under range variations. In summary: (1) key to an optimal performance is the range used and the distance between consecutive tags; (2) the use of MCid technique is recommended over Cid technique; (3) the surveying technique to be used in the determination of the tags’s coordinates should be chosen based on the proposed formulae; (4) the production of tags with the optimal range can significantly reduce the number of tags required to reach E911 standards, compare Tables 2 and 3 where this number is reduced by half. This saves time and costs related to purchase, installation, surveying and maintenance of tags significantly. Therefore the use of the optimal range is recommended.

In the review of literature it was shown that the positioning in urban and indoor areas using RFID based solely on Identification of Cells is feasible (Fu and Retscher 2009; Retscher and Fu 2009) and that the quality of the coordinates determined this way satisfies E911 standards. However, no study reported optimal ranges or smart distributions of tags for location systems as done here. Moreover, the error model considered in these studies is very poor because it does not consider several important parameters as the uncertainty of the tags coordinates, the distance between tags, geometric parameters concerning the environment and range variations. Therefore, the use of the error model proposed here is highly advised.

## Methods

The method employed in our approach to determine error propagation was the mathematical modeling. In other words, the formulae presented in the “Results” section of this article were each mathematically deduced. These mathematical proofs are all presented separately in four appendices.

For reference, we are providing an ODS spreadsheet with the *maper* and *orange* formulae as a shared link at mendeley.com/profiles/eduardo-del-rio, see Del Rio 2011) and also as a Additional file 1. There, their outputs can be studied for any desired instance as its cells may be freely edited by everyone. We included both the direct and indirect maper formulae of each technique in it.

## Appendix 1: Definitions, assumptions and auxiliary relations for the proofs

These appendices are organized as follows: first, we demonstrate the validity of a few relations after defining three functions and stating some assumptions, in a total number of 12 equations—“Appendix 1”; second, the deduction of the *maper* formulae for Cid and MCid techniques follows—“Appendices 2 and 3”, respectively; last, the proof of the *orange* formulae is presented, which is an immediate consequence of the direct *maper* formulae—“Appendix 4”.

Let us define three functions and a set of assumptions to be kept along all of the four Appendices:

24 $$\begin{aligned} \left\{ \begin{array}{l} G(r)=b^2-r\rho -b\sqrt{(r-\rho )^2-l^2} \\ r(b)=\sqrt{\frac{b^2-\rho ^2+l^2}{b^2-\rho ^2}}b \\ F(b,r,\rho ,l)=\sqrt{b^2+(r-\rho )^2-2b\sqrt{(r-\rho )^2-l^2}} \end{array}\right. \end{aligned}$$

where the assumptions are the following: $b\ge \frac{B}{2}, | l | \le L, | \rho  | \le \varepsilon < \frac{B}{2}, \ r \ge L + \varepsilon.$

Based on these functions and on these assumptions, we can deduce a number of equations that will be used to prove the formulae of this article.

25 $$\begin{aligned} \begin{array}{l} \rho > \frac{\sqrt{4b^2+l^2}-l}{2} \Rightarrow G(r)<0, \ \forall r, \ r \ge L+\varepsilon \\ \rho \le \frac{\sqrt{4b^2+l^2}-l}{2} \Rightarrow \left\{ \begin{array}{l} G(r)>0, \ \text{ if } \ \sqrt{\frac{b^2-\rho ^2+l^2}{b^2-\rho ^2}}b > r \ge L+\varepsilon \\ G(r)\le 0, \ \text{ if } \ r \ge \sqrt{\frac{b^2-\rho ^2+l^2}{b^2-\rho ^2}}b \end{array}\right. \end{array} \end{aligned}$$

### *Proof*

*G*(*r*) is decreasing, since its first derivative is negative:

$$\begin{aligned} G'(r)=-\left( \rho +\frac{b(r-\rho )}{\sqrt{(r-\rho )^2-l^2}}\right) \Rightarrow G'(r)<0 \end{aligned}$$

Thus, *G*(*r*) does not have more than 1 zero in the interval $\left[ l+\rho ,\infty \right)$. To determine the sign of *G*(*r*), let us compute the sign of $G(l+\rho )$:

$$\begin{aligned} G(l+\rho )=-\left( \rho +\frac{\sqrt{4b^2+l^2}+l}{2}\right) \left( \rho -\frac{\sqrt{4b^2+l^2}-l}{2}\right) \end{aligned}$$

If $\rho > \frac{\sqrt{4b^2+l^2}-l}{2}$, then $G(l+\rho )$ is negative and therefore *G*(*r*) is also negative for greater values of *r*, since *G*(*r*) is decreasing. On the contrary, $G(l+\rho )$ is positive and thus, *G*(*r*) would be positive for values of *r* lesser than the zero of *G*(*r*) and negative for values of *r* greater than the zero of *G*(*r*). The zero of the function *G*(*r*) is $\sqrt{\frac{b^2-\rho ^2+l^2}{b^2-\rho ^2}}b$.

26 $$\begin{aligned} \rho <\frac{\sqrt{4b^2+l^2}-l}{2} \Rightarrow r'(b)>0 \end{aligned}$$

### *Proof*

Derivating $$r^{2}(b)$$ comes:

$$\begin{aligned} r^{2}(b)=\frac{b^4-\rho ^2 b^2+l^2 b^2}{b^2-\rho ^2} \Rightarrow \end{aligned}$$

$$\begin{aligned} 2r(b)r'(b)=2b\frac{b^4-2\rho ^2 b^2-l^2 \rho ^2+\rho ^4 }{(b^2-\rho ^2)^2} \Rightarrow \end{aligned}$$

$$\begin{aligned} \frac{r(b)(b^2-\rho ^2)^2}{b}r'(b)=-(b^2-\rho ^2+l\rho ) \left( \rho +\frac{\sqrt{4b^2+l^2}+l}{2}\right) \left( \rho -\frac{\sqrt{4b^2+l^2}-l}{2}\right) \end{aligned}$$

Except for the last factor of the previous equation, each factor of the right member is positive, thus concluding the proof.

27 $$\begin{aligned} r(b)>\sqrt{b^2+l^2}-\rho \end{aligned}$$

### *Proof*

Let us define a function *H*(*b*):

$$\begin{aligned} H(b)&=r^{2}(b)-\left( \sqrt{b^2+l^2}-\rho \right) ^2\\ &= \left( r(b)+\left( \sqrt{b^2+l^2}-\rho \right) \right) \left( r(b)-\left( \sqrt{b^2+l^2}-\rho \right) \right) \end{aligned}$$

Factorizing *H*(*b*):

$$\begin{aligned} (b^2-\rho ^2)H(b)&=\rho \left( \rho -\sqrt{b^2+l^2}\right) \left( \rho -\frac{\sqrt{(3b)^2+l^2}+\sqrt{b^2+l^2}}{2}\right) \\ &\quad \times \left( \rho +\frac{\sqrt{(3b)^2+l^2}-\sqrt{b^2+l^2}}{2}\right) \Rightarrow H(b)>0 \end{aligned}$$

28 $$\begin{aligned} \rho <\frac{\sqrt{4b^2+l^2}-l}{2} \Rightarrow r(b)<\sqrt{4b^2+l^2}-\rho \end{aligned}$$

### *Proof*

Let us define a function *I*(*b*):

$$\begin{aligned} I(b)&=r^{2}(b)-\left( \sqrt{4b^2+l^2}-\rho \right) ^2\\ &= \left( r(b)+\left( \sqrt{4b^2+l^2}-\rho \right) \right) \left( r(b)-\left( \sqrt{4b^2+l^2}-\rho \right) \right) \end{aligned}$$

Factorizing *I*(*b*):

$$\begin{aligned} (b^2-\rho ^2)I(b)&=\left( \rho -\frac{\sqrt{4b^2+l^2}+l}{2}\right) \left( \rho -\frac{\sqrt{16b^2+l^2}+\sqrt{4b^2+l^2}}{2}\right) \\& \quad \times  \left( \rho +\frac{\sqrt{16b^2+l^2}+\sqrt{4b^2+l^2}}{2}\right) \left( \rho -\frac{\sqrt{4b^2+l^2}-l}{2}\right) \end{aligned}$$

29 $$\begin{aligned} \rho \le \frac{\sqrt{4b^2+l^2}-l}{2} \Rightarrow r(b)<\sqrt{b^2+l^2}+\rho \end{aligned}$$

### *Proof*

Let us define a function *J*(*b*):

$$\begin{aligned} J(b)=r^{2}(b)-\left( \sqrt{b^2+l^2}+\rho \right) ^2= \left( r(b)+\left( \sqrt{b^2+l^2}+\rho \right) \right) \left( r(b)-\left( \sqrt{b^2+l^2}+\rho \right) \right) \end{aligned}$$

Factorizing *J*(*b*):

$$\begin{aligned} (b^2-\rho ^2)J(b)&=  {} \rho \left( \rho +\sqrt{b^2+l^2}\right) \left( \rho +\frac{\sqrt{(3b)^2+l^2}+\sqrt{b^2+l^2}}{2}\right) \\&\quad \times \left( \rho -\frac{\sqrt{(3b)^2+l^2}-\sqrt{b^2+l^2}}{2}\right) \end{aligned}$$

Once $\rho \le \frac{\sqrt{4b^2+l^2}-l}{2}$, if $\frac{\sqrt{4b^2+l^2}-l}{2} \le \frac{\sqrt{(3b)^2+l^2}-\sqrt{b^2+l^2}}{2}$ then *J*(*b*) is negative. In fact, factorizing

$$\begin{aligned} \left( \sqrt{(3b)^2+l^2}-\sqrt{b^2+l^2}\right) -\left( \sqrt{4b^2+l^2}-l\right) \end{aligned}$$

yields:

$$\begin{aligned} \left( 2b^2+l\sqrt{9b^2+l^2}+\sqrt{4b^2+l^2}\sqrt{b^2+l^2}\right) \left( \sqrt{9b^2+l^2}+\sqrt{b^2+l^2}+\sqrt{4b^2+l^2}+l\right) \\ \left[ \left( \sqrt{(3b)^2+l^2}-\sqrt{b^2+l^2}\right) -\left( \sqrt{4b^2+l^2}-l\right) \right] = 8b^2l\left( l+\sqrt{9b^2+l^2}\right) \end{aligned}$$

Thus proving that $\rho <\frac{\sqrt{(3b)^2+l^2}-\sqrt{b^2+l^2}}{2}$ and *J*(*b*) is negative.

30 $$\begin{aligned} \frac{\partial F(b,r,\rho ,l)}{\partial l}=\frac{bl}{F(b,r,\rho ,l)\sqrt{(r-\rho )^2-l^2}} \end{aligned}$$

### *Proof*

Trivial.

31 $$\begin{aligned} \frac{\partial F(b,r,\rho ,l)}{\partial \rho }= -\frac{r-\rho }{F(b,r,\rho ,l)\sqrt{(r-\rho )^2-l^2}}\left( \sqrt{(r-\rho )^2-l^2}-b\right) \end{aligned}$$

### *Proof*

Trivial.

32 $$\begin{aligned} \frac{\partial F(b,r,\rho ,l)}{\partial r}=-\frac{\partial F(b,r,\rho ,l)}{\partial \rho } \end{aligned}$$

### *Proof*

Trivial.

33 $$\begin{aligned}\left[ r(b)-\left( \sqrt{\left( \frac{2b}{3}\right) ^2+l^2}+\rho \right) \right] \phi& = \left( \rho -\frac{\sqrt{(8b/3)^2+l^2}-\sqrt{(2b/3)^2+l^2}}{2}\right) \nonumber \\    &\quad \times  \left( \rho -\frac{\sqrt{(4b/3)^2+l^2}-\sqrt{(2b/3)^2+l^2}}{2}\right) , \quad \phi >0 \end{aligned}$$

### *Proof*

Let us define the auxiliary functions

$$\begin{aligned} K(b)=\sqrt{\left( \frac{2b}{3}\right) ^2+l^2}+\rho \ \text{ and } \ M(b)=K(b)-\rho \end{aligned}$$

Then:

$$\begin{aligned} 9(b^2-\rho ^2)(r(b)+K(b))(r(b)-K(b))&=  {} \rho ^2(3M(b))^2-6\rho (b^2-\rho ^2)(3M(b))\\ & \quad +5b^4+9\rho ^4-18b^2\rho ^2 \end{aligned}$$

The right member of the previous equation is a second degree polynomial in the variable 3*M*(*b*). Factorizing it:

$$\begin{aligned} 9\rho ^2(b^2-\rho ^2)(r(b)+K(b))(r(b)-K(b))= (3\rho ^2+3M(b)\rho -b^2)(3\rho ^2+3M(b)\rho -5b^2) \end{aligned}$$

Now we have a product of second degree polynomials in $\rho$. Factorizing each one:

$$\begin{aligned} \rho ^2(b^2-\rho ^2)(r(b)+K(b))(r(b)-K(b))&= \left( \rho +\frac{3M(b)+\sqrt{(4b)^2+(3l)^2}}{6}\right) \\& \quad \times  \left( \rho +\frac{3M(b)+\sqrt{(8b)^2+(3l)^2}}{6}\right)& \quad \left( \rho -\frac{\sqrt{(8b/3)^2+l^2}-M(b)}{2}\right) \\ &\quad \times\left( \rho -\frac{\sqrt{(4b/3)^2+l^2}-M(b)}{2}\right) \end{aligned}$$

Concluding the proof.

34 $$\begin{aligned} \left[ r(b)-\left( \sqrt{\left( \frac{2b}{3}\right) ^2+l^2}+\rho \right) \right] \omega = \rho -\frac{\sqrt{(4b/3)^2+l^2}-\sqrt{(2b/3)^2+l^2}}{2}, \nonumber \\ \quad b\ge \frac{3B}{4}, \ \left| l\right| \le L, \ B\ge \sqrt{\frac{64}{105}}L,  \quad \omega <0 \end{aligned}$$

### *Proof*

Starting from Eq. (70), if we show that

$$\begin{aligned} \frac{2b}{3} < \frac{\sqrt{(8b/3)^2+l^2}-\sqrt{(2b/3)^2+l^2}}{2} \end{aligned}$$

under the assumptions considered here, the proof is concluded, since from $b\ge \frac{3B}{4}$ and $\frac{B}{2}>\rho$, it follows: $\rho \le \frac{2b}{3}$. This is done by the following factorization:

$$\begin{aligned} \left( \frac{8b}{3}\sqrt{\left( \frac{2b}{3}\right) +l^2}+\frac{44b^2}{9}\right) \left( \frac{4b}{3}+\sqrt{\left( \frac{8b}{3}\right) ^2+l^2}+\sqrt{\left( \frac{2b}{3}\right) ^2+l^2}\right) \\ \left[ \frac{2b}{3}-\frac{1}{2}\left( \sqrt{\left( \frac{8b}{3}\right) ^2+l^2}-\sqrt{\left( \frac{2b}{3}\right) ^2+l^2}\right) \right] = -\frac{8b^2}{9}\left( \frac{105b^2}{9}-4l^2\right) \end{aligned}$$

35 $$\begin{aligned} \frac{\sqrt{(2b)^2+l^2}-l}{2}<\frac{\sqrt{(8b/3)^2+l^2}-\sqrt{(2b/3)^2+l^2}}{2} \end{aligned}$$

### *Proof*

Let us define the function

$$\begin{aligned} N(b)=\left( \sqrt{(2b)^2+l^2}-l\right) /2- \left( \sqrt{(8b/3)^2+l^2}-\sqrt{(2b/3)^2+l^2}\right) /2 \end{aligned}$$

To show that *N*(*b*) is always negative concludes the proof. This can be done through the following factorization:

$$\begin{aligned} 3\left( \sqrt{(2b)^2+l^2}\sqrt{(2b)^2+(3l)^2}+l\sqrt{(8b)^2+(3l)^2}+4b^2\right) \\ \left( \sqrt{(2b)^2+l^2}+\sqrt{(2b/3)^2+l^2}+\sqrt{(8b/3)^2+l^2}+l\right) \\ N(b)=-\left( 24b^{2}l^{2}+8b^{2}l\sqrt{(8b)^2+(3l)^2}\right) \end{aligned}$$

which concludes the proof.

## Appendix 2: Proof of the cell-id formulae

The error modeling and its related parameters are shown in Fig. 4. The *maper* occurs in one of the following instants: when the user’s RFID reader detects a different tag than the last one he had detected—this last tag might be or not, being detected at this instant; at the very instant immediately before this different tag is detected; when the user’s RFID reader ceases from detecting the last detected tag; at the very instant immediately before this last detected tag ceased from being detected.

In other words, the *maper* is observed at the intersection of the boundary of the surface corresponding to the signal of the last RFID tag detected and the region corresponding to the plane corridor *S*, Figs. 2 and 4. Without loss of generality, we consider that the user’s reader had first detected the tag attached to $\mathbf {X_{n-1}}$ and is now going to detect that of $\mathbf {X_{n}}$. Maintaining the general assumptions of the article, since $\varepsilon$ is the *maper* of the RFID tags positions measurement, let $\rho$, $\rho \le \varepsilon$, be the true error of this measurement and let us assume that when the *maper* is observed the user is at a distance $l, \ \left| l\right| \le L,$ from the line passing through $\mathbf {X_{n-1}X_{n}}$. Angles are referenced to a semi-axis parallel to vector $\mathbf {X_{n-1}X_{n}}$ and measured in a counterclockwise direction. Thus, from Fig. 4:

36 $$\begin{aligned} X_{1}\cos \alpha -X_{2}\cos \beta =B, \ \frac{\pi }{2} \le \beta \le \frac{3\pi }{2} \ \text{ and } -\frac{\pi }{2} \le \alpha \le \frac{3\pi }{2} \end{aligned}$$

where

37 $$\begin{aligned} \left\{ \begin{array}{l} X_{1}\left| \cos \alpha \right| =\sqrt{X_{1}^2-l^2} \\ X_{2}\cos \beta =-\sqrt{X_{2}^2-l^2} \end{array}\right. \end{aligned}$$

The triangle inequality applied to triangle $X_{n}P_{n}X$ yields:

38 $$\begin{aligned} r-\rho \le X_{2} \le r+\rho , \ 0<\rho \le \varepsilon \end{aligned}$$

where the equality occurs if and only if $\mathbf {P_{n}}$, $\mathbf {X}$ and $\mathbf {X_{n}}$ are collinear. Now we will deduce a similar inequality for $X_{1}$ taking Eq. (38) as starting point. Let us consider two cases, each corresponding to a particular interval of range:

**Case a**$$r \le \sqrt{B^2+l^2}-\rho $$

Here, Eq. (38) leads to:

$$\begin{aligned} \left\{ \begin{array}{l} r \le \sqrt{B^2+l^2}-\rho \\ X_{2} \le r+\rho \end{array}\right. \Rightarrow X_{2} \le \sqrt{B^2+l^2} \end{aligned}$$

thus, $-\frac{\pi }{2} \le \alpha \le \frac{\pi }{2}$. Hence, from Eqs. (36) and (37):

39 $$\begin{aligned} -\frac{\pi }{2} \le \alpha \le \frac{\pi }{2} \Rightarrow \sqrt{X_{2}^2-l^2}+\sqrt{X_{1}^2-l^2}=B \end{aligned}$$

From Eq. (38):

$$\begin{aligned} -\sqrt{(r+\rho )^2-l^2} \le -\sqrt{X_{2}^2-l^2} \le -\sqrt{(r-\rho )^2-l^2} \mathop {\Longrightarrow }\limits ^{\hbox {Eq. (39)}} \end{aligned}$$

$$\begin{aligned} B-\sqrt{(r+\rho )^2-l^2} \le \sqrt{X_{1}^2-l^2} \le B-\sqrt{(r-\rho )^2-l^2} \end{aligned}$$

as it is assumed $r \le \sqrt{B^2+l^2}-\rho$, both members of the previous inequality can be squared:

$$\begin{aligned} \left( B-\sqrt{(r+\rho )^2-l^2}\right) ^2+l^2 \le X_{1}^2 \le \left( B-\sqrt{(r-\rho )^2-l^2}\right) ^2+l^2 \Rightarrow \end{aligned}$$

and therefore:

40 $$\begin{aligned}& r \le \sqrt{B^2+l^2}-\rho\Rightarrow & {} \\ & \qquad\sqrt{\left( B-\sqrt{(r+\rho )^2-l^2}\right) ^2+l^2} \le X_{1} \nonumber &\le \sqrt{\left( B-\sqrt{(r-\rho )^2-l^2}\right) ^2+l^2} \end{aligned}$$

**Case b**$$r \ge \sqrt{B^2+l^2}-\rho $$

In this interval of range, two subcases will be considered, each corresponding to a given interval of angle.

**Case b1**$$-\frac{\pi }{2} \le \alpha \le \frac{\pi }{2}$$

For this interval of angles, the Eq. (39) holds. Thus, considering this equation together with Eq. (38), over analogous algebraic manipulations to those of Case a, it follows:

$$\begin{aligned} B-\sqrt{(r+\rho )^2-l^2} \le \sqrt{X_{1}^2-l^2} \le B-\sqrt{(r-\rho )^2-l^2} \end{aligned}$$

and hence:

41 $$\begin{aligned} \left\{ \begin{array}{l} r \ge \sqrt{B^2+l^2}-\rho \\ -\pi /2 \le \alpha \le \pi /2 \end{array}\right. \Rightarrow X_{1} \le \sqrt{\left( B-\sqrt{(r-\rho )^2-l^2}\right) ^2+l^2} \end{aligned}$$

**Case b2**$$\frac{\pi }{2} \le \alpha \le \frac{3\pi }{2}$$

Here, Eqs. (36) and (37) imply:

42 $$\begin{aligned} \frac{\pi }{2} \le \alpha \le \frac{3\pi }{2} \Rightarrow \sqrt{X_{2}^2-l^2}-\sqrt{X_{1}^2-l^2}=B \end{aligned}$$

From Eq. (38):

$$\begin{aligned} \sqrt{(r-\rho )^2-l^2} \le \sqrt{X_{2}^2-l^2} \le \sqrt{(r+\rho )^2-l^2} \mathop {\Longrightarrow }\limits ^{\hbox {Eq. (42)}} \end{aligned}$$

once it had been considered $r \ge \sqrt{B^2+l^2}-\rho$, the right member of the previous inequality can be squared.

43 $$\begin{aligned}&X_{1}^2 \le \left( B-\sqrt{(r+\rho )^2-l^2}\right) ^2+l^2 &\Rightarrow& \nonumber \\&\left\{ \begin{array}{l} r \ge \sqrt{B^2+l^2}-\rho \\ \pi /2 \le \alpha \le 3\pi /2 \end{array}\right. &\Rightarrow& X_{1} \le \sqrt{\left( B-\sqrt{(r+\rho )^2-l^2}\right) ^2+l^2} \end{aligned}$$

Then, Eqs. (41) and (43) lead to:

44 $$\begin{aligned}&r \ge \sqrt{B^2+l^2}-\rho \Rightarrow \nonumber \\&\qquad \qquad X_{1} \le \max \left\{ \sqrt{\left( B-\sqrt{(r-\rho )^2-l^2}\right) ^2+l^2};\right. \left. \sqrt{\left( B-\sqrt{(r+\rho )^2-l^2}\right) ^2+l^2}\right\} \end{aligned}$$

Now, in order to achieve the final expression corresponding to Case b, let us determine the least upper bound for $X_{1}$ from two possibilities, ${\bar{X}}_{1a}$ and ${\bar{X}}_{1b}$:

45 $$\begin{aligned} \left\{ \begin{array}{l} {\bar{X}}_{1a}=\sqrt{\left( B-\sqrt{(r-\rho )^2-l^2}\right) ^2+l^2} \\ {\bar{X}}_{1b}=\sqrt{\left( B-\sqrt{(r+\rho )^2-l^2}\right) ^2+l^2} \end{array}\right. \end{aligned}$$

factorizing $${\bar{X}}_{1a}-{\bar{X}}_{1b}$$:

46 $$\begin{aligned}{\bar{X}}_{1a}-{\bar{X}}_{1b}&=\frac{4}{{\bar{X}}_{1a}+{\bar{X}}_{1b}} \left( \frac{\sqrt{(r+\rho )^2-l^2}-\sqrt{(r-\rho )^2-l^2}}{2B+\sqrt{(r+\rho )^2-l^2}-\sqrt{(r-\rho )^2-l^2}}\right) \\ &\quad\times\left( B^2-r\rho -B\sqrt{(r-\rho )^2-l^2}\right) \end{aligned}$$

Equation (46) can be rewritten as:

47 $$\begin{aligned} \left( {\bar{X}}_{1a}-{\bar{X}}_{1b}\right) \beta =B^2-r\rho -B\sqrt{(r-\rho )^2-l^2}, \ \beta >0 \end{aligned}$$

In the light of Eq. (25), the sign of ${\bar{X}}_{1a}-{\bar{X}}_{1b}$ depends on the magnitude of *B* and on the magnitude of the range, whether *r* is above or beneath *r*(*B*) since *r*(*B*) is always greater than $\sqrt{B^2+l^2}-\rho$ from Eq. (27).

If $B\ge 2L/3$ then $\left( \sqrt{4B^2+l^2}-l\right) /2\ge B/2$ from the following factorization:

$$\begin{aligned} \frac{\sqrt{4B^2+l^2}-l}{2}-\frac{B}{2}=\frac{3B}{2\left( \sqrt{4B^2+l^2}+B+l\right) }\left( B-\frac{2}{3}L\right) \end{aligned}$$

Therefore, denoting the least upper bounds of $X_{1}$ and $X_{2}$ by ${\bar{X}}_{1}$ and ${\bar{X}}_{2}$ respectively, from Eqs. (25, 27, 39) and (47), with $b=B$ where applicable, it follows:

48 $$\begin{aligned} B\ge \frac{2}{3}L \ \text{ and } \ \varepsilon <\frac{B}{2} \Rightarrow \left\{ \begin{array}{l} {\bar{X}}_{1}= \left\{ \begin{array}{l} {\bar{X}}_{1a}=F(B,r,\rho ,l), \ \text{ if } \ l+\rho \le r \le r(B) \\ {\bar{X}}_{1b}=F(B,r,-\rho ,l), \ \text{ if } \ r \ge r(B) \end{array}\right. \\ {\bar{X}}_{2}=r+\rho , \ \text{ if } \ r \ge l+\rho \end{array}\right. \end{aligned}$$

49 $$\begin{aligned} B<\frac{2}{3}L \ \text{ and } \ \frac{\sqrt{4B^2+l^2}-l}{2}<\varepsilon <\frac{B}{2} \Rightarrow \left\{ \begin{array}{l} {\bar{X}}_{1}={\bar{X}}_{1b}=F(B,r,-\rho ,l), \ \text{ if } \ r\ge l+\rho \\ {\bar{X}}_{2}=r+\rho , \ \text{ if } \ r \ge l+\rho \end{array}\right. \end{aligned}$$

50 $$\begin{aligned} B<\frac{2}{3}L \ \text{ and } \ \varepsilon \le \frac{\sqrt{4B^2+l^2}-l}{2} \Rightarrow \left\{ \begin{array}{l} {\bar{X}}_{1}= \left\{ \begin{array}{l} {\bar{X}}_{1a}=F(B,r,\rho ,l), \ \text{ if } \ l+\rho \le r \le r(B) \\ {\bar{X}}_{1b}=F(B,r,-\rho ,l), \ \text{ if } \ r \ge r(B) \end{array}\right. \\ {\bar{X}}_{2}=r+\rho , \ \text{ if } \ r \ge l+\rho \end{array}\right. \end{aligned}$$

Furthermore, since $r(B)<\sqrt{4B^2+L^2}-\varepsilon <L+\varepsilon$ when $\varepsilon >\left( \sqrt{4B^2+L^2}-L\right) /2$, by letting $\rho =\varepsilon$ and $b=B$ in Eq. (28); Eqs. (48–50) can be synthetically expressed by Eq. (4).

The *maper* is the greatest amongst ${\bar{X}}_{1}$ and ${\bar{X}}_{2}$. Factorizing ${\bar{X}}_{1}-{\bar{X}}_{2}$ based on the previous equation:

51 $$\begin{aligned} {\bar{X}}_{1}-{\bar{X}}_{2}= \left\{ \begin{array}{l} \frac{4}{{\bar{X}}_{1a}+{\bar{X}}_{2}}\left( \left( \frac{B}{2}\right) ^2-r\rho -\frac{B}{2}\sqrt{(r-\rho )^2-l^2}\right) , \quad \text{ if } \ {\bar{X}}_{1}={\bar{X}}_{1a} \\ \frac{2B}{{\bar{X}}_{1b}+{\bar{X}}_{2}}\left( \frac{B}{2}-\sqrt{(r+\rho )^2-l^2}\right) ,  \quad\text{ if } \ {\bar{X}}_{1}={\bar{X}}_{1b} \end{array}\right. \end{aligned}$$

If $B\ge 2L/3$ or $\left[ B<2L/3 \ \text{ and } \ \varepsilon \le \left( \sqrt{4B^2+l^2}-l\right) /2\right]$ then the relation of order between ${\bar{X}}_{1}$ and ${\bar{X}}_{2}$ depends on the relation of order between *r* and *r*(*B*). First, if $r \ge r(B)$ then, from Eq. (27), $r \ge \sqrt{B^2+l^2}-\rho$ and hence

$$\begin{aligned} r \ge \sqrt{(B/2)^2+l^2}-\rho \end{aligned}$$

which for instance implies ${\bar{X}}_{1}-{\bar{X}}_{2}<0$ from Eq. (51). Second, if $r \le r(B)$ the sign of ${\bar{X}}_{1}-{\bar{X}}_{2}$ follows from Eq. (25) considering $b=B/2$ on it. Thus, two more cases have to be considered. If $\rho \ge \left( \sqrt{B^2+l^2}-l\right) /2$ then it immediately follows that ${\bar{X}}_{1}-{\bar{X}}_{2}$ is negative. Otherwise, if

$$\begin{aligned} \rho \le \left( \sqrt{B^2+l^2}-l\right) /2 \end{aligned}$$

since $r(B/2)<r(B)$ from Eq. (25), the sign of ${\bar{X}}_{1}-{\bar{X}}_{2}$ will depend whether *r* is above or beneath *r*(*B* / 2).

On the other side, if $B<2L/3$ and $\left( \sqrt{4B^2+l^2}-l\right) /2 \le \varepsilon < B/2$ then $L+\varepsilon \ge \sqrt{4B^2+l^2}-\varepsilon >\sqrt{(B/2)^2+l^2}-\varepsilon$. Since ${\bar{X}}_{1}={\bar{X}}_{1b}$ in the intervals considered, ${\bar{X}}_{2}$ is always greater than ${\bar{X}}_{1}$.

Therefore, from the aforementioned:

52 $$\begin{aligned} \begin{array}{l} \rho \le \frac{\sqrt{B^2+l^2}-l}{2} \Rightarrow \ \left\{ \begin{array}{l} {\bar{X}}_{1}>{\bar{X}}_{2}, \ \text{ if } \ L+\varepsilon \le r \le \sqrt{\frac{(B/2)^2-\rho ^2+l^2}{(B/2)^2-\rho ^2}}\frac{B}{2} \\ {\bar{X}}_{1}={\bar{X}}_{2}, \ \text{ if } \ r=\sqrt{\frac{(B/2)^2-\rho ^2+l^2}{(B/2)^2-\rho ^2}}\frac{B}{2} \\ {\bar{X}}_{1}<{\bar{X}}_{2}, \ \text{ if } \ r > \sqrt{\frac{(B/2)^2-\rho ^2+l^2}{(B/2)^2-\rho ^2}}\frac{B}{2} \end{array}\right. \\ \rho > \frac{\sqrt{B^2+l^2}-l}{2} \Rightarrow {\bar{X}}_{1}<{\bar{X}}_{2}, \ r \ge L+\varepsilon \end{array} \end{aligned}$$

Letting $b\ge B/2$ in Eq. (26), we find that *r*(*b*) is monotonic increasing for $b\ge B/2$ if $\rho \le \left( \sqrt{B^2+l^2}-l\right) /2$–note that $b\ge B/2$ implies

$$\begin{aligned} \left( \sqrt{4b^2+l^2}-l\right) /2\le \left( \sqrt{B^2+l^2}-l\right) /2 \end{aligned}$$

Hence, from Eq. (48) ${\bar{X}}_{1b}$ cannot be the *maper* of Cid, in the light of Eq. (52), as $r(B/2)<r(B)$.

To compute the uncertainty in terms of the *maper* for cid technique it must be determined $\rho$ and *l* for which the error is the greatest. First, if $\rho > \left( \sqrt{B^2+l^2}-l\right) /2$ then the *maper* equals ${\bar{X}}_{2}$ from the previous equation. ${\bar{X}}_{2}$ is monotonic increasing in the variable $\rho$, thus the *maper* is reached for $\rho =\varepsilon$. If $\rho \le \left( \sqrt{B^2+l^2}-l\right) /2$ then the *maper* might be ${\bar{X}}_{1}$ or ${\bar{X}}_{2}$. If it equals ${\bar{X}}_{2}$ the *maper* occurs when $\rho =\varepsilon$. Else, if it equals ${\bar{X}}_{1}$ then $r\le r(B/2)$. From Eq. (29), with $b=B/2$, follows $r(B/2)<\sqrt{(B/2)^2+l^2}+\rho$, and hence $r<\sqrt{(B/2)^2+l^2}+\rho$, which implies from Eq. (31) that ${\bar{X}}_{1a}=F(B,r,\rho ,l)$ is monotonic increasing in the variable $\rho$. $F(B,r,\rho ,l)$ is always monotonic increasing in the variable *l* as a consequence of Eq. (30). Let $\rho _{1} < \varepsilon$ and $l_{1} < L$, then:

$$\begin{aligned} \left\{ \begin{array}{l} F(B,r,\rho _{1},l_{1})<F(B,r,\varepsilon ,l_{1}) \\ F(B,r,\varepsilon ,l_{1})<F(B,r,\varepsilon ,L) \end{array}\right. \Rightarrow \end{aligned}$$

53 $$\begin{aligned} F(B,r,\rho _{1},l_{1})<F(B,r,\varepsilon ,L), \ \forall \rho _{1}<\varepsilon , \ \forall l_{1}<L, r\le r\left(\frac{B}{2}\right) \end{aligned}$$

Thus, the *maper* of Cid technique happens when $\rho =\varepsilon$ and $l=L$; it follows by making these substitutions in Eq. (52), which gives rise to its direct formulas given by Eqs. (4–6).

## Appendix 3: Proof of the mean cell-id formulae

The error modeling and its related parameters are shown in Fig. 5. The instant when the *maper* is observed depends on the number of tags being simultaneously detected. If two tags are never detected simultaneously then MCid is identical to Cid and so it is the occurrence of the *maper*. If at least two tags may be detected at the same time then four possibilities arise: first, no tag is being detected when a distinct tag is detected; second, no more tags are detected when the reader ceases from detecting the last detected tag; third, one tag is being detected at a time when another tag is detected; fourth, two or more tags are being detected at the same time when the reader ceases from detecting one of them. In the first two cases, MCid is equivalent to Cid; thus, the locus of points in which the *maper* is observed is the one described on the beginning of "Appendix 2" for Cid technique. In the third and fourth cases, if two or more tags are being detected at the same time then the *maper* occurs in one of the following instants: at the very instant when the user’s RFID reader ceases from detecting one of the last two tags detected; at the very instant immediately before it ceased from detecting one of the last two tags detected. Otherwise, the *maper* occurs in one of the following instants: at the very instant when the user’s RFID reader turns to two tags being simultaneously detected; at the very instant immediately before it turned to two tags being simultaneously detected.

In other words, if at least two tags are being simultaneously detected, the *maper* is observed at the intersection of the boundary of the surface corresponding to the region simultaneously covered by the signals of the last two RFID tags detected and the region corresponding to the plane corridor *S*, Figs. 2 and 5. Without loss of generality, we consider that the user’s reader had first detected the tag attached to $\mathbf {X_{n-2}}$ and is now going to detect the tags of $\mathbf {X_{n-1}}$ and $\mathbf {X_{n}}$, respectively. Keeping the general assumptions of the Article, $X_{1}$ and $X_{2}$ were both calculated in the previous section and their least upper bounds ${\bar{X}}_{1}$ and ${\bar{X}}_{2}$ are given by Eqs. (48–50). Angles are still referenced to a semi-axis parallel to vector $\mathbf {X_{n-1}X_{n}}$ and are also measured in a counterclockwise direction. Hence, $X_{3}$ follows from Fig. 5:

54 $$\begin{aligned} \left\{ \begin{array}{l} X_{3}\cos \gamma -X_{2}\cos \beta =\frac{B}{2}, \quad \frac{\pi }{2} \le \gamma \le \frac{3\pi }{2}, \ \frac{\pi }{2} \le \beta \le \frac{3\pi }{2} \\ X_{3}\cos \gamma =-\sqrt{X_{3}^2-l^2} \end{array}\right. \end{aligned}$$

Equation (38) yields:

$$\begin{aligned} (r-\rho )^2-l^2 \le X_{2}^2-l^2 \le (r+\rho )^2-l^2 \mathop {\Longrightarrow }\limits ^{(54)} \end{aligned}$$

$$\begin{aligned} \sqrt{(r-\rho )^2-l^2}-\frac{B}{2} \le \sqrt{X_{3}^2-l^2} \le \sqrt{(r+\rho )^2-l^2}-\frac{B}{2} \end{aligned}$$

When $r \ge \sqrt{(B/2)^2+l^2}-\rho$ both members of the previous inequality may be squared. In the interval of $\gamma$ angles considered in Eq. (54) we have $X_{2} \ge \sqrt{(B/2)^2+l^2}$. Then, from Eq. (38):

$$\begin{aligned} \left\{ \begin{array}{l} X_{2} \le r+\rho \\ \sqrt{(B/2)^2+l^2} \le X_{2} \end{array} \right. \Rightarrow r \ge \sqrt{(B/2)^2+l^2}-\rho \end{aligned}$$

Therefore, it follows for $X_{3}$:

55 $$\begin{aligned}&r \ge \sqrt{\left( \frac{B}{2}\right) ^2+l^2}-\rho \Rightarrow \nonumber \\&\qquad \sqrt{\left( \frac{B}{2}-\sqrt{(r-\rho )^2-l^2}\right) ^2+l^2} \le X_{3} \le \sqrt{\left( \frac{B}{2}-\sqrt{(r+\rho )^2-l^2}\right) ^2+l^2} \end{aligned}$$

There is no purpose on the consideration of different intervals for the angle $\gamma$. For such angles, $r<\sqrt{(B/2)^2+l^2}-\rho$ and therefore $X_{3}$ would not be defined because the user’s RFID reader would never detect more than one RFID tag at a given time.

Denoting the least upper bound of $X_{3}$ by ${\bar{X}}_{3}$, there are 4 possibilities for the *maper* of MCid technique: ${\bar{X}}_{1}$, ${\bar{X}}_{2}$, ${\bar{X}}_{3}$ or ${\bar{X}}_{4}$, where ${\bar{X}}_{4}$ is the least upper bound of

$$\begin{aligned} X_{4}=\left\| \mathbf {X}-\left( \mathbf {X_{n-2}}+\mathbf {X_{n-1}}\right) /2\right\| \end{aligned}$$

The expression of ${\bar{X}}_{4}$ is easily determined by making the following substitutions in Cid technique deduction: *B* for 3*B* / 2, $X_{1}$ for $X_{4}$, ${\bar{X}}_{1a}$ for ${\bar{X}}_{4a}$, ${\bar{X}}_{1b}$ for ${\bar{X}}_{4b}$, ${\bar{X}}_{1}$ for ${\bar{X}}_{4}$. Therefore, by letting $\rho =\varepsilon$, $l=L$ and changing *B* for 3*B* / 2 in Eq. (47) follows:

56 $$\begin{aligned} \left( {\bar{X}}_{4a}-{\bar{X}}_{4b}\right) \theta =\left( \frac{3B}{2}\right) ^2-r\varepsilon -\frac{3B}{2}\sqrt{(r-\varepsilon )^2-L^2}, \quad \beta >0 \end{aligned}$$

In the light of Eq. (25), the solution depends on whether $\left( \sqrt{9B^2+L^2}-L\right) /2$, it is greater than or lesser than *B* / 2. This can be determined from the following factorization:

$$\begin{aligned} \frac{\sqrt{9B^2+L^2}-(B+L)}{2}=\frac{B}{\sqrt{9B^2+L^2}+B+L}\left( 4B-L\right) \end{aligned}$$

Therefore:

57 $$\begin{aligned} B\ge \frac{L}{4} \ \text{ and } \ \varepsilon <\frac{B}{2} \Rightarrow {\bar{X}}_{4}= \left\{ \begin{array}{l} {\bar{X}}_{4a}=F(3B/2,r,\varepsilon ,L), \quad \text{ if } \ L+\varepsilon \le r \le r(3B/2) \\ {\bar{X}}_{4b}=F(3B/2,r,-\varepsilon ,L), \quad \text{ if } \ r \ge r(3B/2) \end{array}\right. \end{aligned}$$

58 $$\begin{aligned} B<\frac{L}{4} \ \text{ and } \ \frac{\sqrt{9B^2+L^2}-L}{2}<\varepsilon <\frac{B}{2} \Rightarrow {\bar{X}}_{4}={\bar{X}}_{4b}= F(3B/2,r,-\varepsilon ,L), \quad \text{ if } \ r\ge L+\varepsilon \end{aligned}$$

59 $$\begin{aligned}&B<\frac{L}{4} \ \text{ and } \ \varepsilon \le \frac{\sqrt{9B^2+L^2}-L}{2} \Rightarrow \nonumber \\&\qquad {\bar{X}}_{4}= \left\{ \begin{array}{l} {\bar{X}}_{4a}=F(3B/2,r,\varepsilon ,L), \quad \text{ if } \ L+\varepsilon \le r \le r(3B/2)\\ {\bar{X}}_{4b}=F(3B/2,r,-\varepsilon ,L), \quad \text{ if } \ r \ge r(3B/2) \end{array} \right. \end{aligned}$$

Furthermore, since $r(3B/2)<\sqrt{9B^2+L^2}-\varepsilon <L+\varepsilon$ when $\varepsilon > ( \sqrt{9B^2+L^2}-L) /2$, by letting $\rho =\varepsilon$ and $b=3B/2$ in Eq. (28); Eqs. (57–59) can be synthetically expressed by Eq. (8).

As we have shown in the preceding section, the *maper* of ${\bar{X}}_{1}$ and ${\bar{X}}_{2}$ occurs when $\rho =\varepsilon$ and $l=L$. At the same time, ${\bar{X}}_{4b}$ is not a candidate for the *maper* of MCid technique, as it will be shown along this “Appendix 3”. Consequently, the *maper* of ${\bar{X}}_{4}$ also occurs when $\rho =\varepsilon$ and $l=L$ for its computation is analogous to that of ${\bar{X}}_{1}$. In regard to ${\bar{X}}_{3}$, this is also true, since ${\bar{X}}_{3}=F\left( \frac{B}{2},-r,\rho ,l\right)$. Making $b=\frac{B}{2}$ and substituting *r* by $-r$ in Eq. (30) imply ${\bar{X}}_{3}$ is monotonic increasing in the variable *l*. The same substitutions in Eq. (31) imply ${\bar{X}}_{3}$ is monotonic increasing in the variable $\rho$, since $r \ge \sqrt{(B/2)^2+l^2}-\rho$ (otherwise, ${\bar{X}}_{3}$ is not defined). Let $\rho _{1} < \varepsilon$ and $l_{1} < L$, then:

$$\begin{aligned} \left\{ \begin{array}{l} F(B/2,-r,\rho _{1},l_{1})<F(B/2,-r,\varepsilon ,l_{1}) \\ F(B/2,-r,\varepsilon ,l_{1})<F(B/2,-r,\varepsilon ,L) \end{array}\right. \Rightarrow \end{aligned}$$

60 $$\begin{aligned} F(B/2,-r,\rho _{1},l_{1})<F(B/2,-r,\varepsilon ,L), \forall \rho _{1}<\varepsilon , \ \forall l_{1}<L, r\ge \sqrt{(B/2)^2+l^2}-\rho \end{aligned}$$

Along these lines, it will be shown that only ${\bar{X}}_{4a}$ is a candidate for the *maper* of MCid technique. The *maper* of ${\bar{X}}_{4}$ occurs when $\rho =\varepsilon$ and $l=L$, in an analogous procedure to that used in “Appendix 2” for ${\bar{X}}_{1}$. Thus, the *maper* for MCid technique also occurs when $\rho =\varepsilon$ and $l=L$.

The least upper bound of $X_{3}$, denoted by ${\bar{X}}_{3}$, follows from Eq. (55) and is given by:

61 $$\begin{aligned} {\bar{X}}_{3}=F\left( \frac{B}{2},-r,\varepsilon ,L\right) , \quad \text{ if } \ r \ge \sqrt{\left( \frac{B}{2}\right) ^2+L^2}-\varepsilon \end{aligned}$$

To determine the corresponding *maper* of MCid technique let us consider four cases, each corresponding to a different interval of range.

**Case a**$$L+\varepsilon \le r < \sqrt{\left( \frac{B}{2}\right) ^2+L^2}-\varepsilon $$

In this interval, at most one RFID tag is detected at any given time. Thus, MCid is identical to Cid in this case, which leads to the following *maper*:

62 $$\begin{aligned} \Delta _{MCid}=\max \left\{ {\bar{X}}_{1};{\bar{X}}_{2}\right\} , \quad \text{ if } \ L+\varepsilon \le r < \sqrt{\left( \frac{B}{2}\right) ^2+L^2}-\varepsilon \end{aligned}$$

**Case b**$$\sqrt{\left( \frac{B}{2}\right) ^2+L^2}-\varepsilon \le r < \min \left\{ \sqrt{B^2+L^2}-\varepsilon ;\sqrt{\left( \frac{B}{2}\right) ^2+L^2}+\varepsilon \right\} $$

In this interval, at most two RFID tags may be detected at a given time. Furthermore, the reader will never detects two tags simultaneously at all times, but only at some times. The *maper* in this case is $\max \left\{ {\bar{X}}_{1};{\bar{X}}_{2};{\bar{X}}_{3}\right\}$. However, factorizing ${\bar{X}}_{3}-{\bar{X}}_{2}$ yields:

$$\begin{aligned} {\bar{X}}_{3}-{\bar{X}}_{2}= \frac{B^2}{\left( {\bar{X}}_{3}+{\bar{X}}_{2}\right) \left( (B/2)^2+B\sqrt{(r+\varepsilon )^2-L^2}\right) } \left( \left( \frac{B}{4}\right) ^2-(r+\varepsilon )^2+L^2\right) \end{aligned}$$

once $r \ge \sqrt{\left( \frac{B}{2}\right) ^2+L^2}-\varepsilon$, it also holds $r > \sqrt{\left( \frac{B}{4}\right) ^2+L^2}-\varepsilon$, which implies ${\bar{X}}_{3}-{\bar{X}}_{2}<0$. This way, ${\bar{X}}_{3}$ cannot be the greatest amongst ${\bar{X}}_{1}$, ${\bar{X}}_{2}$ and ${\bar{X}}_{3}$, and hence:

63 $$\begin{aligned} \Delta _{MCid}= & {} \max \left\{ {\bar{X}}_{1};{\bar{X}}_{2}\right\} , \quad \text{ if } \nonumber \\&\sqrt{\left( \frac{B}{2}\right) ^2+L^2}-\varepsilon \le r < \min \left\{ \sqrt{B^2+L^2}-\varepsilon ;\sqrt{\left( \frac{B}{2}\right) ^2+L^2}+\varepsilon \right\}. \end{aligned}$$

Equations (62) and (63) can be joined together in the following equation:

64 $$\begin{aligned} \Delta _{MCid}=\max \left\{ {\bar{X}}_{1};{\bar{X}}_{2}\right\} , \quad \text{ if } L+\varepsilon \le r < \min \left\{ \sqrt{B^2+L^2}-\varepsilon ;\sqrt{\left( \frac{B}{2}\right) ^2+L^2}+\varepsilon \right\} \end{aligned}$$

Solving Eq. (64) by Eq. (52), with $l=L$ and $\rho =\varepsilon$, as $r(\frac{B}{2})<\sqrt{B^2+L^2}-\varepsilon$ and $r(\frac{B}{2})<\sqrt{\left( \frac{B}{2}\right) ^2+L^2}+\varepsilon$, by letting $l=L$ and $\rho =\varepsilon$ in Eqs. (28) and (29) respectively, yields:

65 $$\begin{aligned}&\varepsilon <\frac{\sqrt{B^2+L^2}-L}{2} \Rightarrow \Delta _{MCid}\nonumber \\&\qquad \qquad = \left\{ \begin{array}{l} {\bar{X}}_{1a}, \quad \text{ if } \ L+\varepsilon \le r < r\left(\frac{B}{2}\right) \\ {\bar{X}}_{2}, \quad \text{ if } \ r\left(\frac{B}{2}\right) \le r < \min \left\{ \sqrt{B^2+L^2}-\varepsilon ;\sqrt{\left( \frac{B}{2}\right) ^2+L^2}+\varepsilon \right\} \end{array} \right. \end{aligned}$$

66 $$\begin{aligned} &\frac{B}{2}> {} \varepsilon \ge \frac{\sqrt{B^2+L^2}-L}{2} \Rightarrow \Delta _{MCid}={\bar{X}}_{2},\\ & \qquad\text{ if } \ L+\varepsilon \le r \nonumber < \min \left\{ \sqrt{B^2+L^2}-\varepsilon ;\sqrt{\left( \frac{B}{2}\right) ^2+L^2}+\varepsilon \right\} \end{aligned}$$

**Case c**$$\sqrt{\left( \frac{B}{2}\right) ^2+L^2}+\varepsilon \le r< \sqrt{B^2+L^2}-\varepsilon $$

In this case, the user’s RFID reader will always detect at least one tag. The reader will never detects two tags simultaneously at all times, but only at some times. Thus:

67 $$\begin{aligned} \Delta _{MCid}=\max \left\{ {\bar{X}}_{1};{\bar{X}}_{3}\right\} , \quad \text{ if } \sqrt{\left( \frac{B}{2}\right) ^2+L^2}+\varepsilon \le r< \sqrt{B^2+L^2}-\varepsilon \end{aligned}$$

To factorize ${\bar{X}}_{1}-{\bar{X}}_{3}$, first, if $r\le r(B)$ Eq. (48) with $\rho =\varepsilon$ and $l=L$ yields ${\bar{X}}_{1}-{\bar{X}}_{3}={\bar{X}}_{1a}-{\bar{X}}_{3}$. Since, $r(B)>\sqrt{B^2+L^2}-\varepsilon$ by letting $b=B$, $l=L$ and $\rho =\varepsilon$ in Eq. (27) we always have ${\bar{X}}_{1}-{\bar{X}}_{3}={\bar{X}}_{1a}-{\bar{X}}_{3}$ in this interval of range. Hence:

68 $$\begin{aligned} {\bar{X}}_{1a}-{\bar{X}}_{3}= & {} \frac{B/2+\sqrt{(r+\varepsilon )^2-L^2}-\sqrt{(r-\varepsilon )^2-L^2}}{\left( 3B/2+\sqrt{(r+\varepsilon )^2-L^2}-\sqrt{(r-\varepsilon )^2-L^2}\right) \left( {\bar{X}}_{1a}+{\bar{X}}_{3}\right) } \nonumber \\&\times\left( \left( \frac{3B}{4}\right) ^2-r\rho -\frac{3B}{4}\sqrt{(r-\varepsilon )^2-L^2}\right) \end{aligned}$$

which is equivalent to:

69 $$\begin{aligned} r\le r(B) \Rightarrow {\bar{X}}_{1}-{\bar{X}}_{3}=\left( {\bar{X}}_{1a}-{\bar{X}}_{3}\right) \beta = \left( \frac{3B}{4}\right) ^2-r\varepsilon -\frac{3B}{4}\sqrt{(r-\varepsilon )^2-L^2}, \ \beta >0 \end{aligned}$$

By letting $b=3B/4$ in Eq. (25), it can be verified that the sign of ${\bar{X}}_{1a}-{\bar{X}}_{3}$ depends on: the magnitude of $\varepsilon$, whether it is above or beneath $\left( \sqrt{(3B/2)^2+L^2}-L\right) /2$. Furthermore, still from Eq. (25), this sign may depend on the magnitude of *r*, whether it is above or beneath *r*(3*B* / 4). First of all, let us determine the relation of order between:

$$\begin{aligned} \left( \sqrt{(3B/2)^2+L^2}-L\right) /2 \ \text{ and } \ B/2 \end{aligned}$$

which is given by the following equation

70 $$\begin{aligned} \frac{8}{5B}\left( \sqrt{(3B/2)^2+L^2}+B+L\right) \left( \frac{\sqrt{(3B/2)^2+L^2}-L}{2}-\frac{B}{2}\right) =B-\frac{8}{5}L \end{aligned}$$

Therefore, to determine the uncertainty in the light of the *maper* for MCid technique in this interval of range, let us consider two additional subcases:

**Case c1**$$B>\frac{8}{5}L$$

Equation (70) implies $( \sqrt{(3B/2)^2+L^2}-L) /2>B/2$. Thus, $\varepsilon <( \sqrt{(3B/2)^2+L^2}-L)$ for these values of *B*. From Eq. (25), with $b=3B/4$, $\rho =\varepsilon$ and $l=L$, the sign of ${\bar{X}}_{1}-{\bar{X}}_{3}$ depends on the magnitude of *r*.

If $r(3B/4)<\sqrt{(B/2)^2+L^2}+\varepsilon$ then ${\bar{X}}_{1}-{\bar{X}}_{3}$ is always negative. Otherwise, the signal of ${\bar{X}}_{1}-{\bar{X}}_{3}$ is ruled by the relation of order between *r* and *r*(3*B* / 4).

Here, it is assumed $B>8L/5$. As $8L/5>\sqrt{64/105}L$, it follows that $B>\sqrt{64/105}L$. Letting $b=3B/4$, $\rho =\varepsilon$ and $l=L$ in Eqs. (25) and (34), the *maper* arises—in this Case c, it is assumed that $r\ge \sqrt{(B/2)^2+L^2}+\varepsilon$ and this must be taken into account:

71 $$\begin{aligned}&B>\frac{8}{5}L \ \text{ and } \ \varepsilon \le \frac{\sqrt{B^2+L^2}-\sqrt{(B/2)^2+L^2}}{2} \Rightarrow \nonumber \\&\qquad \qquad \Delta _{MCid}= \left\{ \begin{array}{l} {\bar{X}}_{1a}, \ \text{ if } \ \sqrt{\left( \frac{B}{2}\right) ^2+L^2}+\varepsilon \le r \le r\left( \frac{3B}{4}\right) \\ {\bar{X}}_{3}, \ \text{ if } \ \sqrt{B^2+L^2}-\varepsilon \ge r> r\left( \frac{3B}{4}\right) \end{array}\right. \end{aligned}$$

72 $$\begin{aligned}&B>\frac{8}{5}L \ \text{ and } \ \frac{B}{2} > \varepsilon > \frac{\sqrt{B^2+L^2}-\sqrt{(B/2)^2+L^2}}{2} \Rightarrow \nonumber \\&\qquad \qquad \Delta _{MCid}={\bar{X}}_{3}, \ \text{ if } \ \sqrt{B^2+L^2}-\varepsilon \ge r \ge \sqrt{\left( \frac{B}{2}\right) ^2+L^2}+\varepsilon \end{aligned}$$

**Case c2**$$B\le \frac{8}{5}L$$

$$B\le \frac{8}{5}L$$ implies $$\left( \sqrt{(3B/2)^2+L^2}-L\right) /2\le B/2$$.

If $\left( \sqrt{(3B/2)^2+L^2}-L\right) /2<\varepsilon <B/2$ then the *maper* follows immediately from Eq. (25) with $b=3B/4$, $\rho =\varepsilon$ and $l=L$:

73 $$\begin{aligned} B\le \frac{8}{5}L \ \text{ and } \ \frac{B}{2} > \varepsilon > \frac{\sqrt{(3B/2)^2+L^2}-L}{2} \Rightarrow \Delta _{MCid}={\bar{X}}_{3}, \ \text{ if } \ r \ge \sqrt{\left( \frac{B}{2}\right) ^2+L^2}+\varepsilon \end{aligned}$$

Now, if $\varepsilon \le ( \sqrt{(3B/2)^2+L^2}-L) /2$ then the *maper* depends on the magnitude of *r* and whether *r*(3*B* / 4) is greater than or lesser than $\sqrt{(B/2)^2+L^2}+\varepsilon$.

$L<\sqrt{(B/2)^2+L^2}$ implies $-L>-\sqrt{(B/2)^2+L^2}$ and, since $\sqrt{(3B/2)^2+L^2}>\sqrt{B^2+L^2}$, it follows from addition and subsequent division by 2:

$${{\left( {\sqrt {(3B/2)^{2}  + L^{2} }-L} \right)} \mathord{\left/ {\vphantom {{\left( {\sqrt {(3B/2)^{2}  + L^{2} }-L} \right)} 2}} \right.} 2}>{{\left( {\sqrt {(B/2)^{2}  + L^{2} }  - \sqrt {B^{2}  + L^{2} } } \right)} \mathord{\left/ {\vphantom {{\left( {\sqrt {(B/2)^{2}  + L^{2} }  - \sqrt {B^{2}  + L^{2} } } \right)} 2}} \right.} 2}$$

From Eq. (35), also with $b=3B/4$:

$${{\left( {\sqrt {(3B/2)^{2}  + L^{2} }  - L} \right)} \mathord{\left/ {\vphantom {{\left( {\sqrt {(3B/2)^{2}  + L^{2} }  - L} \right)} 2}} \right.} 2} < {{\left( {\sqrt {(2B)^{2}  + L^{2} }  - \sqrt {(B/2)^{2}  + L^{2} } } \right)} \mathord{\left/ {\vphantom {{\left( {\sqrt {(2B)^{2}  + L^{2} }  - \sqrt {(B/2)^{2}  + L^{2} } } \right)} 2}} \right.} 2}$$

Therefore, the *maper* will be derived from two possibilities:

$$\begin{aligned} &{{\varepsilon  \le \left( {\sqrt {B^{2}  + L^{2} }  - \sqrt {(B/2)^{2}  + L^{2} } } \right)} \mathord{\left/ {\vphantom {{\varepsilon  \le \left( {\sqrt {B^{2}  + L^{2} }  - \sqrt {(B/2)^{2}  + L^{2} } } \right)} 2}} \right.}2} \hfill \\  & {\text{ and }}{{\left( {\sqrt {B^{2}  + L^{2} }  - \sqrt {(B/2)^{2}  + L^{2} } } \right)} \mathord{\left/ {\vphantom {{\left( {\sqrt {B^{2}  + L^{2} }  - \sqrt {(B/2)^{2}  + L^{2} } } \right)} 2}} \right.} 2} < \varepsilon  \le {{\left( {\sqrt {(3B/2)^{2}  + L^{2} }  - L} \right)} \mathord{\left/ {\vphantom {{\left( {\sqrt {(3B/2)^{2}  + L^{2} }  - L} \right)} 2}} \right.} 2} \hfill \\  \end{aligned}  $$

The *maper* when $\varepsilon \le \left( \sqrt{(3B/2)^2+L^2}-L\right) /2$ is achieved by letting $l=L$, $\rho =\varepsilon$ and $b=3B/4$ in Eqs. (25) and (33):

74 $$\begin{aligned}&B\le \frac{8}{5}L \ \text{ and } \ \frac{\sqrt{(3B/2)^2+L^2}-L}{2}\ge \varepsilon \ge \frac{\sqrt{B^2+L^2}-\sqrt{(B/2)^2+L^2}}{2} \Rightarrow \nonumber \\&\qquad \qquad \Delta _{MCid}={\bar{X}}_{3}, \ \text{ if } \ \sqrt{B^2+L^2}-\varepsilon \ge r \ge \sqrt{\left( \frac{B}{2}\right) ^2+L^2}+\varepsilon \end{aligned}$$

75 $$\begin{aligned}&B\le \frac{8}{5}L \ \text{ and } \ \varepsilon \le \frac{\sqrt{B^2+L^2}-\sqrt{(B/2)^2+L^2}}{2} \Rightarrow \nonumber \\&\qquad \qquad \Delta _{MCid}= \left\{ \begin{array}{l} {\bar{X}}_{1a}, \ \text{ if } \ \sqrt{\left( \frac{B}{2}\right) ^2+L^2}+\varepsilon \le r \le r\left( \frac{3B}{4}\right) \\ {\bar{X}}_{3}, \ \text{ if } \ \sqrt{B^2+L^2}-\varepsilon \ge r> r\left( \frac{3B}{4}\right) \end{array} \right. \end{aligned}$$

Therefore the *maper* for subcase c2 is given by two equations: the first being obtained by joining Eqs. (73) and (94) into the the following equation:

76 $$\begin{aligned}&B\le \frac{8}{5}L \ \text{ and } \ \frac{B}{2}\ge \varepsilon \ge \frac{\sqrt{B^2+L^2}-\sqrt{(B/2)^2+L^2}}{2} \Rightarrow \nonumber \\&\qquad \Delta _{MCid}={\bar{X}}_{3}, \ \text{ if } \ \sqrt{B^2+L^2}-\varepsilon \ge r \ge \sqrt{\left( \frac{B}{2}\right) ^2+L^2}+\varepsilon \end{aligned}$$

The second corresponding to Eq. (75) itself. Thus, the *maper* for subcase c2 is given by Eqs. (75) and (76).

Finally, the *maper* corresponding to Case c follows from Eqs.  (71, 72, 75) and (76):

77 $$\begin{aligned} \varepsilon \le \frac{\sqrt{B^2+L^2}-\sqrt{(B/2)^2+L^2}}{2} \Rightarrow \Delta _{MCid}= \left\{ \begin{array}{l} {\bar{X}}_{1a}, \ \text{ if } \ \sqrt{\left( \frac{B}{2}\right) ^2+L^2}+\varepsilon \le r \le r\left( \frac{3B}{4}\right) \\ {\bar{X}}_{3}, \ \text{ if } \ \sqrt{B^2+L^2}-\varepsilon >r> r\left( \frac{3B}{4}\right) \end{array}\right. \end{aligned}$$

78 $$\begin{aligned}&\frac{B}{2} > \varepsilon > \frac{\sqrt{B^2+L^2}-\sqrt{(B/2)^2+L^2}}{2} \Rightarrow \nonumber \\&\qquad \qquad \Delta _{MCid}={\bar{X}}_{3}, \ \text{ if } \ \sqrt{B^2+L^2}-\varepsilon >r \ge \sqrt{\left( \frac{B}{2}\right) ^2+L^2}+\varepsilon \end{aligned}$$

**Case d**$$r\ge \sqrt{B^2+L^2}-\varepsilon $$

For this interval of ranges the reader always detect at least one RFID tag at any given time. But it may occur that it detects two RFID tags at any given time. Therefore, three possibilities arise for the *maper* in this case: ${\bar{X}}_{1}$, ${\bar{X}}_{3}$ and ${\bar{X}}_{4}$.

We will now show that in this particular interval of range $\max \left\{ {\bar{X}}_{1};{\bar{X}}_{3};{\bar{X}}_{4}\right\} =\max \left\{ {\bar{X}}_{3};{\bar{X}}_{4}\right\}$. To do this, let us consider two possibilities: $-\pi /2\le \alpha \le \pi /2$ or $\pi /2\le \alpha \le 3\pi /2$.

First, if $\pi /2\le \alpha \le 3\pi /2$ then Eq. (42) and Eq. (54) imply:

$$\begin{aligned} \sqrt{X_{2}^2-L^2}=B+\sqrt{X_{1}^2-L^2}=\frac{B}{2}+\sqrt{X_{3}^2-L^2} \Rightarrow \end{aligned}$$

$$\begin{aligned} \sqrt{X_{1}^2-L^2}=\sqrt{X_{3}^2-L^2}-\frac{B}{2} \Rightarrow \sqrt{X_{1}^2-L^2}< \sqrt{X_{3}^2-L^2} \end{aligned}$$

thus:

79 $$\begin{aligned} \pi /2\le \alpha \le 3\pi /2 \Rightarrow X_{1} \le X_{3} \end{aligned}$$

As a consequence, $${\bar{X}}_{1}\le {\bar{X}}_{3}$$.

On the other side, if $-\pi /2\le \alpha \le \pi /2$ then, from Eq. (39), $\sqrt{X_{2}^2-L^2}\le B$ and:

$$\begin{aligned} \sqrt{X_{2}^2-L^2}\le B \mathop {\Longrightarrow }\limits ^{\hbox {Eq. (49)}} \frac{3B}{2}-\sqrt{X_{4}^2-L^2}\le B \Rightarrow X_{4}\ge \sqrt{(B/2)^2+L^2} \end{aligned}$$

since the deduction of $X_{4}$ is analogous to that of $X_{1}$.

Concurrently, Eq. (39) for $X_{1}$ and Eq. (54) for $X_{3}$, imply:

$$\begin{aligned} \sqrt{X_{2}^2-L^2}=B-\sqrt{X_{1}^2-L^2}=\frac{B}{2}+\sqrt{X_{3}^2-L^2} \Rightarrow \end{aligned}$$

$$\begin{aligned} \sqrt{X_{1}^2-L^2}+\sqrt{X_{3}^2-L^2}=\frac{B}{2} \Rightarrow X_{1} \le \sqrt{(B/2)^2+L^2} \end{aligned}$$

thus:

80 $$\begin{aligned} -\pi /2\le \alpha \le \pi /2 \Rightarrow X_{1} \le X_{4} \end{aligned}$$

Therefore, if $-\pi /2\le \alpha \le \pi /2$ then $X_{1}\le X_{4}$. Consequently, ${\bar{X}}_{1}\le {\bar{X}}_{4}$. This way, from Eqs. (79) and (80), ${\bar{X}}_{1}$ can be removed from the expression for the *maper* of MCid in this interval of range.

81 $$\begin{aligned} \varepsilon < \frac{B}{2} \Rightarrow \Delta _{MCid}=\max \left\{ {\bar{X}}_{3};{\bar{X}}_{4}\right\} , \ \text{ if } \ r\ge \sqrt{B^2+L^2}-\varepsilon \end{aligned}$$

Now, to analytically calculate $\max \left\{ {\bar{X}}_{3};{\bar{X}}_{4}\right\}$, let us factorize ${\bar{X}}_{3}-{\bar{X}}_{4}$:

$$\begin{aligned} {\bar{X}}_{3}-{\bar{X}}_{4a}&= -4\frac{B+\sqrt{(r+\varepsilon )^2-L^2}+\sqrt{(r-\varepsilon )^2-L^2}}{\left( {\bar{X}}_{3}+{\bar{X}}_{4a}\right) \left( 2B+\sqrt{(r+\varepsilon )^2-L^2}-\sqrt{(r-\varepsilon )^2-L^2}\right) } \\ &\quad\times\left( B^2-r\varepsilon -B\sqrt{(r-\varepsilon )^2-L^2}\right) \end{aligned}$$

which implies:

82 $$\begin{aligned} \left( {\bar{X}}_{3}-{\bar{X}}_{4a}\right) \theta =B^2-r\varepsilon -B\sqrt{(r-\varepsilon )^2-L^2}, \ \theta <0 \end{aligned}$$

In case $${\bar{X}}_{4}={\bar{X}}_{4b}$$:

83 $$\begin{aligned} {\bar{X}}_{3}-{\bar{X}}_{4b}=\frac{-2B}{{\bar{X}}_{3}+{\bar{X}}_{4b}}\left( B-\sqrt{(r+\varepsilon )^2-L^2}\right) \end{aligned}$$

which means that ${\bar{X}}_{3}$ is always greater than ${\bar{X}}_{4b}$ if

$$\begin{aligned} r\ge \sqrt{B^2+L^2}-\varepsilon \end{aligned}$$

For this reason, ${\bar{X}}_{4b}$ cannot be the *maper* of MCid technique.

To determine $\max \left\{ {\bar{X}}_{3};{\bar{X}}_{4}\right\}$ from Eqs. (57), (82) and (25), let us consider three subcases:

**Case d1**$$B\ge 2L/3$$

Here, since $r(3B/2)>r(B)$, from Eq. (26), and $r(B)>\sqrt{B^2+L^2}-\varepsilon$, from Eq. (27), it follows:

84 $$\begin{aligned} B\ge 2L/3 \ \text{ and } \ \varepsilon <B/2 \Rightarrow \Delta _{MCid}= \left\{ \begin{array}{l} {\bar{X}}_{4a}, \ \text{ if } \ \sqrt{B^2+L^2}-\varepsilon \le r < r(B) \\ {\bar{X}}_{3}, \ \text{ if } \ r \ge r(B) \end{array} \right. \end{aligned}$$

**Case d2**$$2L/3>B\ge L/4$$

85 $$\begin{aligned}&2L/3>B\ge L/4 \ \text{ and } \ \varepsilon <\left( \sqrt{4B^2+L^2}-L\right) /2 \Rightarrow \nonumber \\&\qquad \qquad \Delta _{MCid}= \left\{ \begin{array}{l} {\bar{X}}_{4a}, \ \text{ if } \ \sqrt{B^2+L^2}-\varepsilon \le r < r(B) \\ {\bar{X}}_{3}, \ \text{ if } \ r \ge r(B) \end{array}\right. \end{aligned}$$

86 $$\begin{aligned}&2L/3>B\ge L/4 \ \text{ and } \ \left( \sqrt{4B^2+L^2}-L\right) /2\le \varepsilon <B/2 \Rightarrow \nonumber \\&\qquad \quad \Delta _{MCid}= {\bar{X}}_{3}, \ \text{ if } \ r \ge \sqrt{B^2+L^2}-\varepsilon \end{aligned}$$

**Case d3**$$B<L/4$$

87 $$\begin{aligned} B<L/4 \ \text{ and } \ \left( \sqrt{9B^2+L^2}-L\right) /2\le \varepsilon <B/2 \Rightarrow \Delta _{MCid}= {\bar{X}}_{3}, \ \text{ if } \ r \ge \sqrt{B^2+L^2}-\varepsilon \end{aligned}$$

88 $$\begin{aligned}&B<L/4 \ \text{ and } \ \left( \sqrt{4B^2+L^2}-L\right) /2\le \varepsilon <\left( \sqrt{9B^2+L^2}-L\right) /2 \Rightarrow \nonumber \\&\qquad \Delta _{MCid}= {\bar{X}}_{3}, \ \text{ if } \ r \ge \sqrt{B^2+L^2}-\varepsilon \end{aligned}$$

89 $$\begin{aligned}&B<L/4 \ \text{ and } \ \varepsilon <\left( \sqrt{4B^2+L^2}-L\right) /2 \Rightarrow \Delta _{MCid}\nonumber \\&\qquad \qquad = \left\{ \begin{array}{l} {\bar{X}}_{4a}, \ \text{ if } \ \sqrt{B^2+L^2}-\varepsilon \le r < r(B) \\ {\bar{X}}_{3}, \ \text{ if } \ r \ge r(B) \end{array} \right. \end{aligned}$$

From Eqs. (84–89), the *maper* of Case d is finally achieved:

90 $$\begin{aligned} B\ge 2L/3 \ \text{ and } \ \varepsilon <B/2 \Rightarrow \Delta _{MCid}= \left\{ \begin{array}{l} {\bar{X}}_{4a}, \ \text{ if } \ \sqrt{B^2+L^2}-\varepsilon \le r < r(B) \\ {\bar{X}}_{3}, \ \text{ if } \ r \ge r(B) \end{array}\right. \end{aligned}$$

91 $$\begin{aligned}&B<2L/3 \ \text{ and } \ \varepsilon <\left( \sqrt{4B^2+L^2}-L\right) /2 \Rightarrow \Delta _{MCid}\nonumber \\&\qquad \qquad = \left\{ \begin{array}{l} {\bar{X}}_{4a}, \ \text{ if } \ \sqrt{B^2+L^2}-\varepsilon \le r < r(B) \\ {\bar{X}}_{3}, \ \text{ if } \ r \ge r(B) \end{array}\right. \end{aligned}$$

92 $$\begin{aligned}&B<2L/3 \ \text{ and } \ \left( \sqrt{4B^2+L^2}-L\right) /2\le \varepsilon <B/2 \Rightarrow \Delta _{MCid}\nonumber \\&\qquad \qquad = \left\{ \begin{array}{l} {\bar{X}}_{3}, \ \text{ if } \ r \ge \sqrt{B^2+L^2}-\varepsilon \end{array} \right. \end{aligned}$$

From Eqs. (65, 66, 77, 78, 90, 91) and (92) the uncertainty of MCid in the light of the *maper* concept is achieved.

93 $$\begin{aligned}&\varepsilon \le \frac{\sqrt{B^2+L^2}-\sqrt{(B/2)^2+L^2}}{2} \Rightarrow \nonumber \\&\qquad \Delta _{MCid}= \left\{ \begin{array}{l} {\bar{X}}_{1a}, \ \text{ if } \ L+\varepsilon \le r < r(\frac{B}{2}) \\ {\bar{X}}_{2}, \ \text{ if } \ r(\frac{B}{2}) \le r < \min \left\{ \sqrt{B^2+L^2}-\varepsilon ;\sqrt{(B/2)^2+L^2}+\varepsilon \right\} \\ {\bar{X}}_{1a}, \ \text{ if } \ \sqrt{\left( \frac{B}{2}\right) ^2+L^2}+\varepsilon \le r \le r\left( \frac{3B}{4}\right) \\ {\bar{X}}_{3}, \ \text{ if } \ r\left( \frac{3B}{4}\right) \le r < \sqrt{B^2+L^2}-\varepsilon \\ {\bar{X}}_{4a}, \ \text{ if } \ \sqrt{B^2+L^2}-\varepsilon \le r < r\left( B\right) \\ {\bar{X}}_{3}, \ \text{ if } \ r\ge r\left( B\right) \end{array} \right. \end{aligned}$$

94 $$\begin{aligned}&\frac{\sqrt{B^2+L^2}-L}{2} > \varepsilon > \frac{\sqrt{B^2+L^2}-\sqrt{(B/2)^2+L^2}}{2} \Rightarrow \nonumber \\&\qquad \Delta _{MCid}= \left\{ \begin{array}{l} {\bar{X}}_{1a}, \ \text{ if } \ L+\varepsilon \le r < r\left( \frac{B}{2}\right) \\ {\bar{X}}_{2}, \ \text{ if } \ r\left( \frac{B}{2}\right) \le r < \min \left\{ \sqrt{B^2+L^2}-\varepsilon ;\sqrt{(B/2)^2+L^2}+\varepsilon \right\} \\ {\bar{X}}_{3}, \ \text{ if } \ \sqrt{\left( \frac{B}{2}\right) ^2+L^2}+\varepsilon \le r < \sqrt{B^2+L^2}-\varepsilon \\ {\bar{X}}_{4a}, \ \text{ if } \ \sqrt{B^2+L^2}-\varepsilon \le r < r\left( B\right) \\ {\bar{X}}_{3}, \ \text{ if } \ r\ge r\left( B\right) \end{array} \right. \end{aligned}$$

95 $$\begin{aligned}&B\ge \frac{2}{3}L \ \text{ and } \ \frac{B}{2} > \varepsilon > \frac{\sqrt{B^2+L^2}-L}{2} \Rightarrow \nonumber \\ &\qquad \qquad \Delta _{MCid}= \left\{ \begin{array}{l} {\bar{X}}_{2}, \ \text{ if } \ L+\varepsilon \le r <\min \left\{ \sqrt{B^2+L^2}-\varepsilon ;\sqrt{(B/2)^2+L^2}+\varepsilon \right\} \\ {\bar{X}}_{3}, \ \text{ if } \ \sqrt{\left( \frac{B}{2}\right) ^2+L^2}+\varepsilon \le r < \sqrt{B^2+L^2}-\varepsilon \\ {\bar{X}}_{4a}, \ \text{ if } \ \sqrt{B^2+L^2}-\varepsilon \le r < r\left( B\right) \\ {\bar{X}}_{3}, \ \text{ if } \ r\ge r\left( B\right) \end{array} \right. \end{aligned}$$

96 $$\begin{aligned}&B< \frac{2}{3}L \ \text{ and } \ \frac{\sqrt{4B^2+L^2}-L}{2} > \varepsilon > \frac{\sqrt{B^2+L^2}-L}{2} \Rightarrow \nonumber \\&\qquad \Delta _{MCid}= \left\{ \begin{array}{l} {\bar{X}}_{2}, \ \text{ if } \ L+\varepsilon \le r <\min \left\{ \sqrt{B^2+L^2}-\varepsilon ;\sqrt{(B/2)^2+L^2}+\varepsilon \right\} \\ {\bar{X}}_{3}, \ \text{ if } \ \sqrt{\left( \frac{B}{2}\right) ^2+L^2}+\varepsilon \le r < \sqrt{B^2+L^2}-\varepsilon \\ {\bar{X}}_{4a}, \ \text{ if } \ \sqrt{B^2+L^2}-\varepsilon \le r < r\left( B\right) \\ {\bar{X}}_{3}, \ \text{ if } \ r\ge r\left( B\right) \end{array}\right. \end{aligned}$$

97 $$\begin{aligned}&B< \frac{2}{3}L \ \text{ and } \ \frac{B}{2} > \varepsilon > \frac{\sqrt{4B^2+L^2}-L}{2} \Rightarrow \nonumber \\&\qquad \Delta _{MCid}= \left\{ \begin{array}{l} {\bar{X}}_{2}, \ \text{ if } \ L+\varepsilon \le r <\min \left\{ \sqrt{B^2+L^2}-\varepsilon ;\sqrt{(B/2)^2+L^2}+\varepsilon \right\} \\ {\bar{X}}_{3}, \ \text{ if } \ r\ge \sqrt{\left( \frac{B}{2}\right) ^2+L^2}+\varepsilon \end{array} \right. \end{aligned}$$

where ${\bar{X}}_{1a}$ and ${\bar{X}}_{2}$ are given by Eqs. (48–50) with $\rho =\varepsilon$ and $l=L$; ${\bar{X}}_{3}$ is given by Eq. (61); ${\bar{X}}_{4a}$ is given by Eqs. (57–59) $\rho =\varepsilon$ and $l=L$; $r=\sqrt{R^2-h^2}$.

Last, the aforementioned expressions can be simplified by the following inequalities:

98 $$\begin{aligned} \varepsilon > \frac{\sqrt{B^2+L^2}-\sqrt{(B/2)^2+L^2}}{2} \Rightarrow \sqrt{(B/2)^2+L^2}+\varepsilon >\sqrt{B^2+L^2}-\varepsilon \end{aligned}$$

99 $$\begin{aligned} \min \left\{ \frac{B}{2};\frac{\sqrt{4B^2+L^2}-L}{2}\right\} >\varepsilon > \frac{\sqrt{B^2+L^2}-L}{2} \Rightarrow L+\varepsilon >\sqrt{B^2+L^2}-\varepsilon \end{aligned}$$

100 $$\begin{aligned} \frac{B}{2} > \varepsilon > \frac{\sqrt{4B^2+L^2}-L}{2} \Rightarrow L+\varepsilon >\sqrt{4B^2+L^2}-\varepsilon >\sqrt{B^2+L^2}-\varepsilon \end{aligned}$$

Applying Eq. (98) to Eqs. (93, 94), Eq. (99) to Eqs. (95, 96), and Eq. (100) to Eq. (97); the final direct formulae for the *maper* of MCid technique are obtained which are given by Eqs. (8–12).

## Appendix 4: Proof of the *orange* formulae

The oranges for Cid and MCid techniques are a natural consequence of the analytic *maper* formulae. They follow from the behavior of function $F(B,r,\varepsilon ,L)$ with respect to the variable *r*, which is ruled by Eq. (32), letting $\rho =\varepsilon$ and $l=L$ in it. Given that ${\bar{X}}_{1a}=F(B,r,-\varepsilon ,L)$, ${\bar{X}}_{1a}$ is monotonic decreasing if $r<\sqrt{B^2+L^2}-\varepsilon$ and increasing otherwise. Equivalently: ${\bar{X}}_{3}=F(B/2,r,-\varepsilon ,L)$ implies that ${\bar{X}}_{3}$ is monotonic increasing if $r>\sqrt{(B/2)^2+L^2}-\varepsilon$; ${\bar{X}}_{4a}=F(3B/2,r,-\varepsilon ,L)$ implies that ${\bar{X}}_{4a}$ is monotonic decreasing if

$$\begin{aligned} r<\sqrt{(3B/2)^2+L^2}-\varepsilon \end{aligned}$$

and increasing otherwise. For ${\bar{X}}_{2}$ it is evident that it is always monotonic increasing since ${\bar{X}}_{2}=r+\varepsilon$.

The *orange* formulae are determined by analyzing the local behavior of the *maper* functions $\Delta _{Cid}$ and $\Delta _{MCid}$ for each interval of uncertainty $\varepsilon$ in particular. From the *r* for which the *maper* reaches its minimal, the *orange**R* of the technique in consideration follows, as $R=\sqrt{r^2+h^2}$. The *orange* determination is self-evident except for $r_{MCid}$ in the specific case when

$$\begin{aligned} \left( \sqrt{B^2+L^2}-L\right) /2 > \varepsilon > \left( \sqrt{B^2+L^2}-\sqrt{(B/2)^2+L^2}\right) /2 \end{aligned}$$

In this situation, the analysis of the function $\Delta _{MCid}$’s behavior in regard to *r* leads us towards two possibilities for $r_{MCid}$: *r*(*B*/2) and *r*(*B*). Let us show that $r_{MCid}$ in this case is *r*(*B*). For this purpose, we have to prove that ${\bar{X}}_{4a}\left( r=r(B)\right) < {\bar{X}}_{1a}\left( r=r(B/2)\right)$. Given that ${\bar{X}}_{4a}\left( r=r(B)\right) ={\bar{X}}_{3}\left( r=r(B)\right)$ and ${\bar{X}}_{1a}\left( r=r(B/2)\right) ={\bar{X}}_{2}\left( r=r(B/2)\right)$, this is equivalent to prove that ${\bar{X}}_{3}\left( r=r(B)\right) < {\bar{X}}_{2}\left( r=r(B/2)\right)$.

To do so, let us determine the sign of ${\bar{X}}_{2}\left( r=r(B/2)\right) -{\bar{X}}_{3}\left( r=r(B)\right)$. From Eqs. (38) to (55), with $\rho =\varepsilon$ and $l=L$, it follows for ${\bar{X}}_{2}\left( r=r(B/2)\right)$ and ${\bar{X}}_{3}\left( r=r(B)\right)$:

$$\begin{aligned} \left\{ \begin{array}{l} \sqrt{\left[ {\bar{X}}_{2}\left( r=r(B/2)\right) \right] ^2-L^2}=\sqrt{\left( r(B/2)+\varepsilon \right) ^2-L^2} \\ -\sqrt{\left[ {\bar{X}}_{3}\left( r=r(B)\right) \right] ^2-L^2}=B/2- \sqrt{\left( r(B)+\varepsilon \right) ^2-L^2} \end{array} \right. \Rightarrow \end{aligned}$$

101 $$\begin{aligned}&\sqrt{\left[ {\bar{X}}_{2}\left( r=r(B/2)\right) \right] ^2-L^2}-\sqrt{\left[ {\bar{X}}_{3}\left( r=r(B)\right) \right] ^2-L^2}=\nonumber \\&\qquad \frac{B}{2}+\sqrt{\left( r(B/2)+\varepsilon \right) ^2-L^2}-\sqrt{\left( r(B)+\varepsilon \right) ^2-L^2} \end{aligned}$$

But the right side of the inequality above can be factorized as follows:

$$\begin{aligned}&\left( \frac{B}{2}+\sqrt{\left( r(B/2)+\varepsilon \right) ^2-L^2}+\sqrt{\left( r(B)+\varepsilon \right) ^2-L^2}\right)& \\ &\times\left( \frac{B}{2}+\sqrt{\left( r(B/2)+\varepsilon \right) ^2-L^2}-\sqrt{\left( r(B)+\varepsilon \right) ^2-L^2}\right)& \\&\quad=\frac{B^2}{4}+\left( r(B/2)+\varepsilon \right) ^2+B\sqrt{\left( r(B/2)+\varepsilon \right) ^2-L^2}-\left( r(B)+\varepsilon \right)& \end{aligned}$$

once the previous equation’s right side is positive, Eq. (101) implies ${\bar{X}}_{3}\left( r=r(B)\right) <{\bar{X}}_{2}\left( r=r(B/2)\right)$.

This way, when

$$\begin{aligned} \left( \sqrt{B^2+L^2}-L\right) /2 > \varepsilon > \left( \sqrt{B^2+L^2}-\sqrt{(B/2)^2+L^2}\right) /2 \end{aligned}$$

the Orange is $r_{MCid}=r(B)$.

## Additional file

Additional file 1: Spreadsheet with maper and orange formulae.
